# Ten new species of *Lophodermium* (*Rhytismatales*, *Rhytismataceae*) on pine needles in China

**DOI:** 10.3897/imafungus.17.175730

**Published:** 2026-01-20

**Authors:** Shi-Juan Wang, Lan Zhuo, Xin-Yi Xu, Xiao-Nan Sui, Xiao-Ye Shen, Yuan-Yuan Li, Cheng-Lin Hou

**Affiliations:** 1 School of Forestry & Landscape Architecture, Anhui Agricultural University, West Changjiang Road 130, Hefei, Anhui 230036, China Capital Normal University Beijing China; 2 Anhui Provincial Key Laboratory of Biological Control, Anhui Agricultural University, West Changjiang Road 130, Hefei, Anhui 230036, China Institute of Medicinal Plant Development, Chinese Academy of Medical Sciences & Peking Union Medical College Beijing China; 3 College of Life Science, Capital Normal University, Xisanhuanbeilu 105, Haidian District, Beijing 100048, China Anhui Provincial Key Laboratory of Biological Control, Anhui Agricultural University Hefei China; 4 Institute of Medicinal Plant Development, Chinese Academy of Medical Sciences & Peking Union Medical College, Beijing 100193, China School of Forestry & Landscape Architecture, Anhui Agricultural University Hefei China

**Keywords:** *
Lophodermium
*, *
Pinus
*, Phylogeny, Taxonomy

## Abstract

*Lophodermium*, the largest genus within the family *Rhytismataceae*, comprises diverse plant-associated endophytes and pathogens, including species responsible for serious diseases that cause substantial economic losses. Pine trees (*Pinus* spp.), among the most species-rich and widely distributed conifers worldwide, serve as hosts for many *Lophodermium* species. In the present study, ten new species of *Lophodermium* are identified and described from pine needles in China, based on an integrated approach combining morphological characteristics and multi-gene phylogenetic analyses. The discovery of these new species significantly expands the known diversity of *Lophodermium* spp. and offers important insights into host specificity and geographic distribution. Furthermore, this work provides an essential scientific foundation for monitoring and managing *Lophodermium*-associated diseases in pine forests.

## Introduction

*Lophodermium* is a relatively large and complex genus within the *Rhytismataceae*, comprising more than 160 known species (Kirk et al. 2008; Index Fungorum 2025). It is widely distributed across most temperate and tropical regions worldwide. Species of *Lophodermium* exhibit broad host ranges, are reported from numerous plant families, and are particularly common as endophytes in conifers such as *Abies*, *Picea*, and *Pinus* (Cannon and Minter 1983; Johnston 1989, 1992, 2001; Ellis and Ellis 1997; Stone et al. 2000). In most *Lophodermium* species associated with pines, ascomata usually develop on recently shed needles. These fungi colonize healthy needles, reside as endophytes within symptomless host tissues, then ascomata typically mature after needle abscission (Deckert et al. 2001). Several species within the genus, such as *L. confluens*, *L. seditiosum*, and others, are pathogens responsible for needle cast diseases (Minter and Millar 1980; Sinclair et al. 1987; Lin et al. 1995). Recent phylogenetic studies have revealed that *Lophodermium* is polyphyletic, with known species scattered across multiple clades of the phylogenetic tree (Ortiz García et al. 2003; Lantz et al. 2011; Zhuo et al. 2025). As knowledge of *Lophodermium* species diversity remains incomplete, corresponding taxonomic conclusions have not yet been drawn. As in previous publications, our study is also based on this broad concept of the genus.

The genus *Pinus (Pinaceae)* comprises more than 100 currently recognized species, making it the largest extant genus of conifers (Price et al. 1998; Farjon 2001). Pines are ecologically significant as major, often dominant, components of Arctic, subalpine, temperate, and tropical forests, as well as arid woodlands (Richardson and Rundel 1998). Economically, they are vital sources of timber, pulp, resin, charcoal, food (notably seeds), and ornamental plants (Gernandt et al. 2005). As the core distribution area of *Pinus* in East Asia, China harbors rich lineages of pine species (Liu et al. 2022; Yue et al. 2025). Among all known *Lophodermium* species, 39 (Index Fungorum 2025; Theron et al. 2025) are reported from pine needles, with 16 species recorded first in China (Lin 2012; Li et al. 2016; Salas-Lizana and Onno 2018; Ata et al. 2024).

Building on this background, the present study focuses on *Lophodermium* species occurring on *Pinus* needles in China. Employing an integrated approach combining multi-gene phylogenetic analyses and morphological characteristics, ten new species are recognized and molecular sequences for two known species, *Lophodermium yuexiense* and *Ploioderma pini-armandii*, are included in the tree. These results contribute significantly to the understanding of *Lophodermium* diversity on needles of trees in the *Pinaceae*.

## Materials and methods

### Specimen collection and isolation

Fresh specimens were collected in China. Specimens were air-dried, placed in paper bags, and stored in a cool, dry location in the laboratory for subsequent studies. Ascomata were cut from the twigs of conifers and disinfected in 75% ethanol for 30 s, followed by 10% sodium hypochlorite (NaOCl) for 3 min, washed in sterile water three times, then placed on Petri dishes containing potato dextrose agar (PDA) and incubated at room temperature (20 °C). Hyphae emerging from the surface of the ascomata were isolated and subcultured on individual PDA plates. Living cultures of new species from this study were deposited in Capital Normal University Culture Collection Center (**CNUCC**) in China.

### Morphological studies

Mature ascomata were selected for morphological analyses. External shape, size, color, and opening of the ascomata and conidiomata, as well as characteristics of zone lines and other details were observed and photographed using a Nikon SMZ-1000 stereomicroscope (Japan). For a detailed description of methods for the morphological analysis, see Wang et al. (2023) and Guo et al. (2024). Dry specimens were deposited at the Herbarium of the College of Life Science, Capital Normal University (**BJTC**). New names have been registered in the MycoBank database (http://www.mycobank.org/).

### Molecular techniques

Genomic DNA was extracted from specimens and cultures with the M5 Plant Genomic DNA Kit (Mei5 Biotechnology Co., Ltd., China) following the manufacturer’s instructions. The ITS regions were amplified with PCR using the primers ITS1f/ITS4 (White et al. 1990; Gardes and Bruns 1993), LR0R/LR5 primers were used for nrLSU (White et al. 1990), and mrSSU1/mrSSU3R primers were used for mtSSU (Zoller et al. 1999). PCR was performed in 25 µL reactions according to previous studies (Guo et al. 2024, 2025; Zhuo et al. 2025). The PCR products were sent to Zhongkexilin Biotechnology Co., Ltd. (Beijing, China) for purification, sequencing, and editing.

### Phylogenetic analysis

The forward and reverse DNA sequences were aligned to generate consensus sequences using SeqMan v.7.1.0 in the DNASTAR Lasergene Core Suite software (DNASTAR Inc., Madison, WI, USA). The newly obtained sequences were submitted to the GenBank database, and additional ITS, nrLSU, and mtSSU sequences included in this study were downloaded from GenBank (Suppl. material 3). The analysis includes type and representative species from all known genera with available molecular data within *Cudoniaceae*, *Rhytismataceae* s.s, and *Triblidiaceae*. For polyphyletic genera within *Rhytismataceae* s.l., such as *Coccomyces* De Not. and *Lophodermium* Chevall., a representative species from each clade of these genera was selected. *Pezicula carpinea* (Pers.) Tul. & C. Tul. ex Fuckel (*Helotiales*, *Dermateaceae*) and *Cudoniella clavus* (Alb. & Schwein.) Dennis (*Helotiales*, *Tricladiaceae*) were selected as outgroups based on Lantz et al. (2011) and Guo et al. (2024). The ITS, nrLSU, and mtSSU datasets were aligned with MAFFT (https://www.ebi.ac.uk/Tools/msa/mafft/), and then manually corrected by eye in Se-Al v.2.03a (Rambaut 2000). Ambiguously aligned regions were not used in the analysis. A combined dataset of ITS, nrLSU, and mtSSU sequences was prepared and analyzed using the maximum parsimony method performed with PAUP* 4.0b10 (Swofford 1998). Maximum parsimony analysis was conducted using heuristic searches with 1,000 replicates of random-addition sequences, TBR branch swapping, and no maxtree limit. All characteristics were equally weighted and unordered. Gaps were treated as missing data to minimize homology assumptions. A bootstrap analysis was performed with 1,000 replicates, each with 100 random taxon addition sequences. MAXTREES was set to 1,000, and TBR branch swapping was employed. For the BI analysis, MrModeltest 2.3 with the Akaike information criterion (AIC) was used to choose the substitution model for each gene: GTR+I+G for ITS, GTR+I+G for nrLSU, and GTR+I+G for mtSSU. The Bayesian analysis was performed with MrBayes 3.1.2 (Huelsenbeck et al. 2001; Ronquist and Huelsenbeck 2003). The analysis of four chains was conducted for 100,000,000 generations with the default settings and sampled every 100 generations, halting the analysis at an average standard deviation of split frequencies of 0.01. The first 25% of the trees were removed as burn-in. PP were obtained from the 50% majority rule consensus of the remaining trees. Maximum likelihood (ML) analysis was performed with IQ-TREE 2.2.0 (Minh et al. 2020), the substitution model for ITS is TIM2e+I+R5, for nrLSU is TIM3e+R4, and for mtSSU is TVM+F+I+R4. ML bootstrap replicates (1000) were computed in IQ-TREE using a rapid bootstrap analysis and search for the best-scoring ML tree. We only considered clades supported by bootstrap values (MLB) ≥ 70% for the ML analysis, supported by bootstrap values (MPB) ≥ 70% for the MP analysis and supported by PP ≥ 0.95 for Bayesian inference.

## Results

### Molecular phylogeny

**Figure 1.**
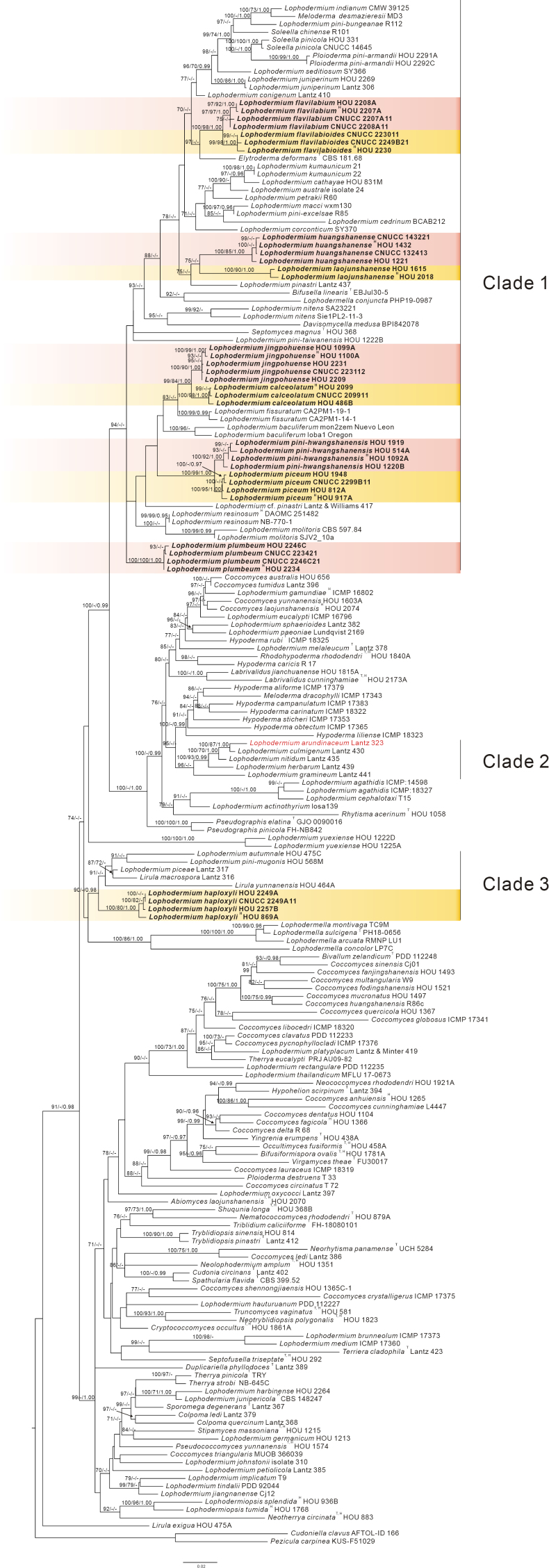
Phylogenetic tree derived from maximum likelihood analysis of combined ITS, nrLSU, and mtSSU rDNA sequences of *Rhytismatales*, using *Cudoniella clavus* (AFTOL-ID 166) and *Pezicula carpinea* (KUS-F51029) as outgroups. Bootstrap support values for ML analysis (MLB) and MP analysis (MPB) greater than 70% and Bayesian posterior probabilities (PP) greater than 0.95 are given above the nodes. Names of new species and a new combination are written in bold; the name of the type species of the genus *Lophodermium* is written in red. ^T^ = type species; ^H^ = holotype.

Sequences obtained from newly collected specimens of *Lophodermium* spp. on pine needles in the present study, together with sequences of species of *Rhytismatales* retrieved from GenBank, were used to construct the dataset for phylogenetic analysis (Suppl. material 3). After removing ambiguously aligned regions, the combined matrix included 2074 base positions of which 867 were parsimony-informative. The maximum parsimony analysis of sequences resulted in one most parsimonious tree (Fig. 1) with a length (TL) of 7347 steps, consistency index (CI) of 0.257, retention index (RI) of 0.601, and homoplasy index (HI) of 0.743. The single loci phylogenetic trees of ITS (Suppl. material 1) and the nrLSU-mtSSU phylogenetic tree (Suppl. material 2) show topologies similar to those of the ITS-nrLSU-mtSSU phylogenetic tree.

The sequence data of new species provided in this work are located in ten lineages within two clades (clades 1 and 3; Fig. 1), namely, *Lophodermium calceolatum*, *L. flavilabium*, *L. flavilabioides*, *L. huangshanense*, *L. jingpohuense*, *L. laojunshanense*, *L. piceum*, *L. pini-hwangshanensis*, and *L. plumbeum* in clade 1 and *L. haploxyli* in clade 3. None of the new sequences cluster with the sequence of the type species, *Lophodermium arundinaceum* in clade 2. For some species, sequences were obtained both directly from specimens and from cultures derived from the same specimens; minor differences of one or a few nucleotides were observed in a few cases but did not affect species delimitation or phylogenetic placement.

### Taxonomy

#### Lophodermium calceolatum

Taxon classificationFungiRhytismatalesRhytismataceae

L. Zhuo & C.L. Hou sp. nov.

9EE9339D-940F-5DEA-B5A3-021E957BE187

861483

Figs 2, 3

##### Diagnosis.

This new species differs from *Lophodermium jingpohuense* by the absence of lip cells and by a covering stroma that is incurved at the opening.

**Figure 2.**
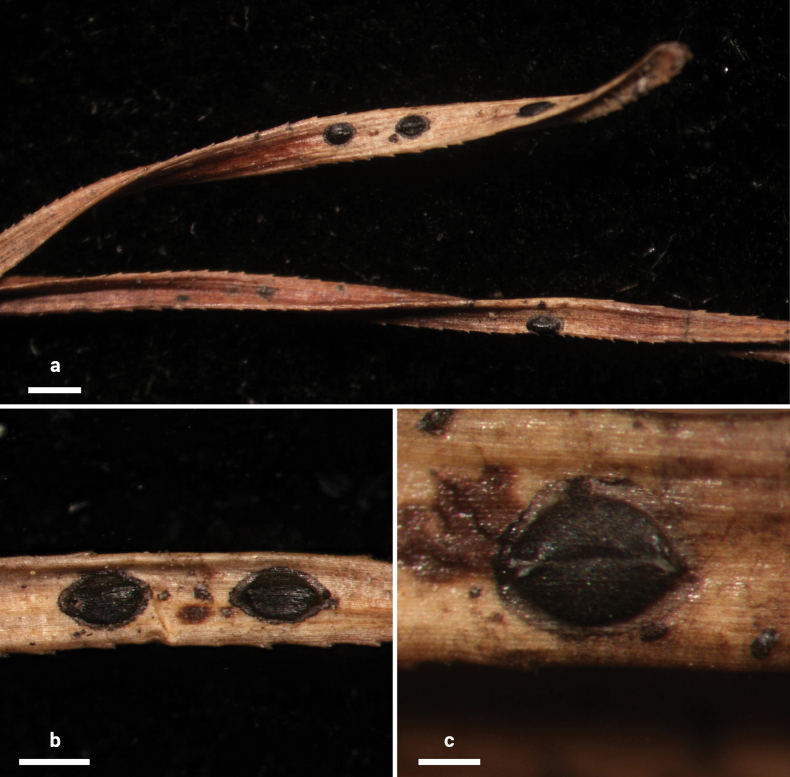
*Lophodermium calceolatum* on *Pinus armandii* (HOU 2099/BJTC 2023229, holotype). **a** Ascomata on needles. **b, c** Mature ascomata. Scale bars: 1 mm (**a**); 500 μm (**b**); 200 μm (**c**).

**Figure 3.**
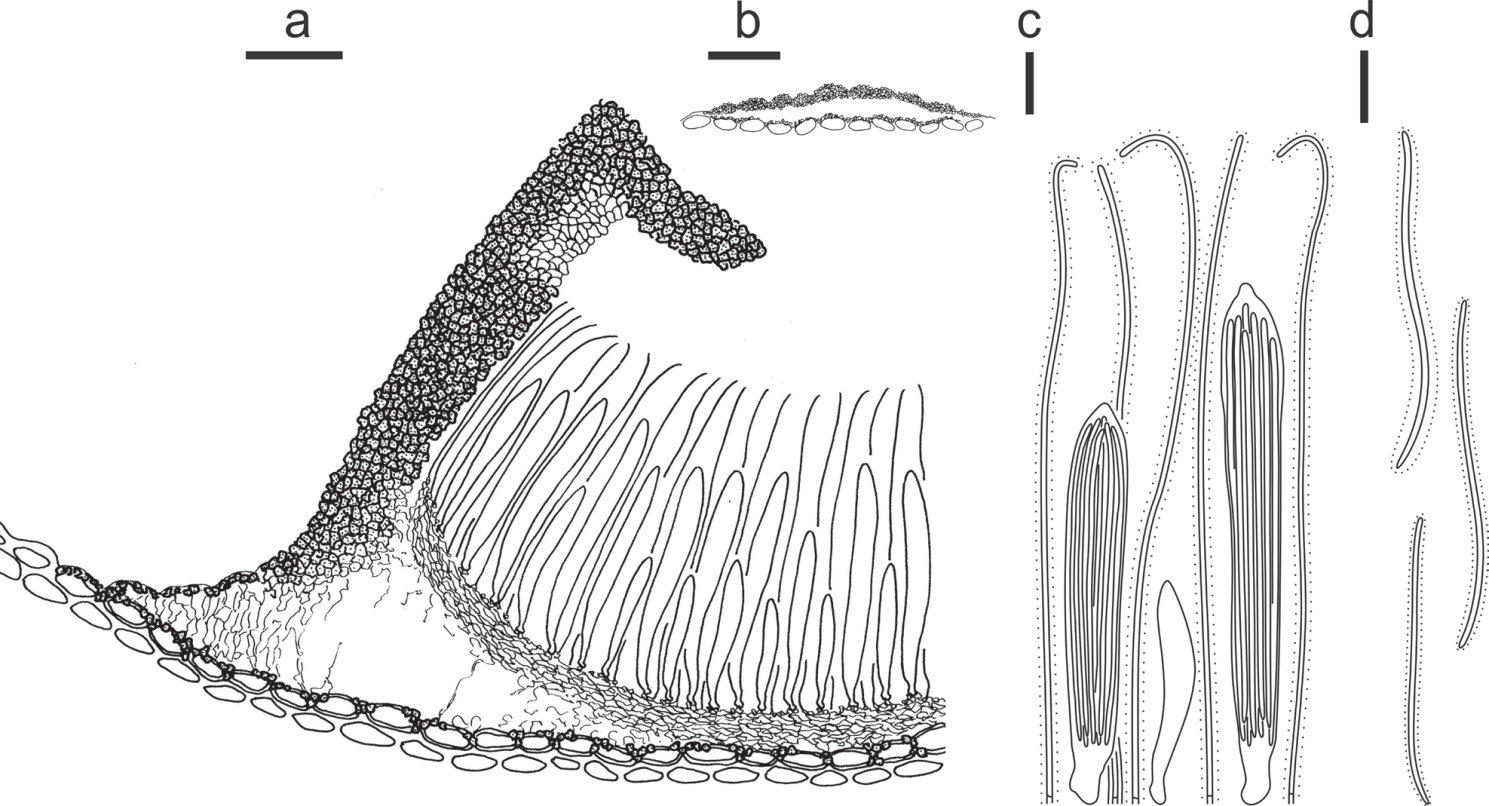
*Lophodermium calceolatum* on *Pinus armandii* (HOU 2099/BJTC 2023229, holotype). **a** Part of an ascoma in vertical section. **b** Conidioma in vertical section. **c** Paraphyses, mature asci with ascospores, and immature ascus. **d** Liberated ascospores. Scale bars: 30 μm (**a**); 100 μm (**b**); 10 μm (**c, d**).

##### Type.

CHINA • Yunnan Province, Lijiang, Laojunshan, 26°38'36"N, 99°46'04"E, ca. 3550 m, on needles of *Pinus armandii* Franch. (*Pinaceae*), 17 Aug 2023, *C.L. Hou, L. Zhuo & S.Y. Zhao*, HOU 2099 (**holotype**BJTC 2023229).

##### Etymology.

*calceolatus* (Latin) = slipper-like, referring to the shape of the ascomata in vertical section.

##### Description.

**Sexual morph: *Ascomata*** mostly on abaxial surface of needles, scattered, associated with pale areas. In surface view, ascomata elliptical, 600–1100 × 350–500 µm, black in center with a gray border and a black perimeter line, opening by a single longitudinal split. ***Lips*** absent. In median vertical section, ascomata subcuticular. ***Covering stroma*** at the opening incurved at a right angle, 30–50 μm thick near center of ascomata, not extending to basal stroma or with only a thin tissue extension, consisting of an outer layer of host cuticle and an inner layer of carbonized, thick-walled angular cells. Near opening, a distinct hyaline zone (30–40 × 20–30 µm) of covering stroma formed by hyaline, thin-walled, angular cells. ***Basal stroma*** poorly developed, consisting of carbonized, thick-walled, angular cells. A space triangular in vertical section between the covering stroma and basal stroma at margin of ascoma filled with hyaline textura prismatica and fragmented fungal tissues. ***Subhymenium*** 15–20 µm thick, consisting of hyaline textura porrecta. ***Paraphyses*** aseptate, filiform, not branched, not swollen at tips, slightly curved, 95–105 × 1 µm, covered by ca. 2 μm thick gelatinous sheaths. ***Asci*** ripening sequentially, cylindrical, pointed at apex, 45–90 × 8–10 µm, thin-walled, J–, 8-spored. ***Ascospores*** aseptate, filiform, 40–80 × 1 μm, hyaline, covered by 1–2 μm thick gelatinous sheaths.

**Asexual morph: *Conidiomata*** scattered, elliptical to irregular, 180–300 × 100–150 µm diam., dark brown to black, opening by an ostiole. In vertical section, conidiomata subcuticular. ***Upper layer*** 15–25 μm thick, consisting of host cuticle and carbonized angular to globose cells. ***Basal layer*** poorly developed. ***Conidiogenous cells*** and ***conidia*** not seen. ***Zone lines*** not seen.

##### Additional specimen examined.

CHINA • Yunnan Province, Lijiang, Tiejiashan, ca. 2000 m, on needles of *P. armandii*, 11 Jul 2007, *C.L. Hou*, HOU 486B (BJTC 2007050).

##### Distribution.

Known only from Yunnan Province, China.

##### Notes.

In the phylogenetic tree, sequences of *L. calceolatum* form a sister clade with *L. jingpohuense*, but the latter species has lip cells and covering stroma is not incurved with a right angle at the opening. Morphologically, *L. calceolatum* closely resembles *L. fissuratum* Salas-Lizana & Oono in sharing the incurved covering stroma (Salas-Lizana and Onno 2018). However, the latter differs by swollen paraphysis tips, longer asci (88–101 μm), and ascospores (140–160 μm). The sequence similarity of ITS rDNA between *L. calceolatum* and *L. fissuratum* is 93%, indicating that *L. calceolatum* is a distinct species.

#### Lophodermium flavilabium

Taxon classificationFungiRhytismatalesRhytismataceae

L. Zhuo & C.L. Hou sp. nov.

C96C4495-12AE-509A-A476-0D676628BEC3

861630

Figs 4, 5

##### Diagnosis.

This new species differs from *Lophodermium flavilabioides* by more conspicuous lips embedded in a gelatinous matrix and paraphyses that are not branched at their tips.

**Figure 4.**
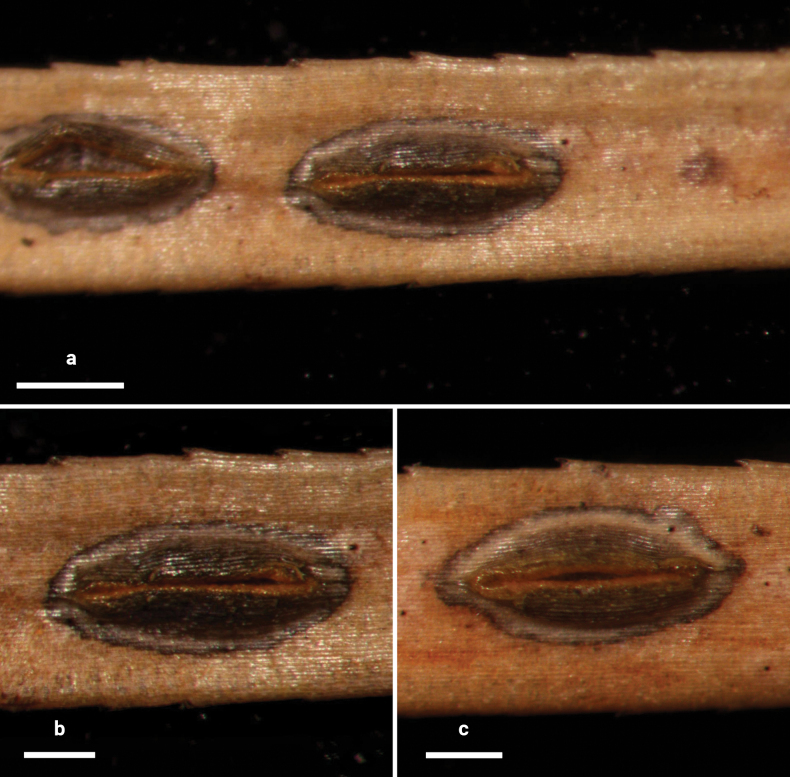
*Lophodermium flavilabium* on *Pinus koraiensis* (HOU 2207A/BJTC 2024067, holotype). **a** Ascomata on needles. **b, c** Mature ascomata and conidiomata. Scale bars: 500 μm (**a**); 300 μm (**b, c**).

**Figure 5.**
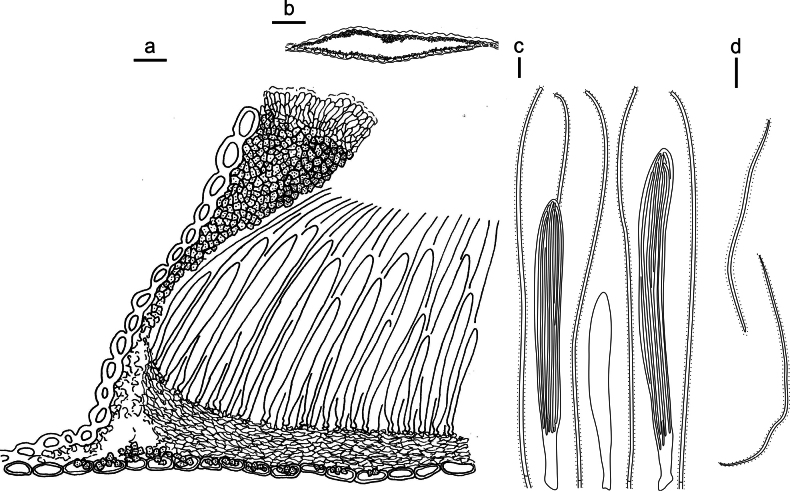
*Lophodermium flavilabium* on *Pinus koraiensis* (HOU 2207A/BJTC 2024067, holotype). **a** Part of an ascoma in vertical section. **b** Conidioma in vertical section. **c** Paraphyses, mature asci with ascospores, and immature ascus. **d** Liberated ascospores. Scale bars: 20 μm (**a**); 200 μm (**b**); 10 μm (**c, d**).

##### Type.

CHINA • Jilin Province, Yanbian Chaoxianzu Autonomous Prefecture, Changbaishan, 42°31'37"N, 128°16'09"E, ca. 640 m, on needles of *Pinus koraiensis* Siebold & Zucc. (*Pinaceae*), 14 Jun 2024, *C.L. Hou, L. Zhuo & Y. Gao*, HOU 2207A (**holotype**BJTC 2024067).

##### Etymology.

*flavi-* (Latin) = yellow, *labium* (Latin) = lips, referring to the yellow lips of this species.

##### Description.

**Sexual morph: *Ascomata*** mostly on abaxial surface of needles, scattered, not associated with pale areas. In surface view, ascomata elliptical, 950–1750 × 325–550 µm, dark gray to black in center with a gray border and brown perimeter line, opening by a single longitudinal split. ***Lips*** yellow to orange. In median vertical section, ascomata completely subepidermal. ***Covering stroma*** 50–70 μm thick near center of ascomata, not extending to basal stroma, consisting of an outer layer of host cuticle and an inner layer of carbonized, thick-walled angular cells. ***Lip cells*** cylindrical to clavate, 12–15 × 2–4 μm, with hyaline base and yellow to orange pigmented cellular contents at apex, embedded in a gelatinous matrix. ***Basal stroma*** poorly developed, consisting of carbonized, thick-walled, angular cells. ***Subhymenium*** 10–18 µm thick, of hyaline textura porrecta. ***Paraphyses*** aseptate, filiform, not branched, not swollen at their tips, 150–180 × 1 µm. ***Asci*** ripening sequentially, cylindrical to clavate, somewhat pointed at apex, 130–160 × 10–15 µm, thin-walled, J–, 8-spored. ***Ascospores*** aseptate, filiform, 60–120 × 1 μm, hyaline, covered by 1–2 μm thick gelatinous sheaths.

**Asexual morph: *Conidiomata*** scattered, round, 180–300 μm diam., concolorous with needle surface, opening by an ostiole. In vertical section, conidiomata subepidermal. ***Upper layer*** 15–30 μm thick, consisting of host cuticle and carbonized angular to globose cells. ***Basal layer*** poorly developed. ***Conidiogenous cells*** and ***conidia*** not seen. ***Zone lines*** not seen.

##### Additional specimen examined.

CHINA • Jilin Province, Yanbian Chaoxianzu Autonomous Prefecture, Changbaishan, 42°31'34"N, 128°16'09"E, ca. 660 m, on needles of *P. koraiensis*, 14 Jun 2024, *C.L. Hou, L. Zhuo & Y. Gao*, HOU 2208A (BJTC 2024068).

##### Distribution.

Known from Jilin Province and Heilongjiang Province, China.

##### Notes.

In the phylogenetic tree, sequences of *L. flavilabium* form a sister clade with *L. flavilabioides*. Morphologically, this species possesses paler-colored ascomata and yellow lips embedded in a gelatinous matrix and can be distinguished from other *Lophodermium* species on *Pinus* subgen. *Strobus* except *L. flavilabioides*. *Lophodermium flavilabioides* differs from *L. flavilabium* by paler lips and paraphyses with branched tips.

#### Lophodermium flavilabioides

Taxon classificationFungiRhytismatalesRhytismataceae

L. Zhuo & C.L. Hou sp. nov.

81D557A1-23DD-5818-8057-F59C03187B2B

861654

Figs 6, 7

##### Diagnosis.

This new species can be distinguished from *Lophodermium flavilabium* by the paraphyses branched at their tips.

**Figure 6.**
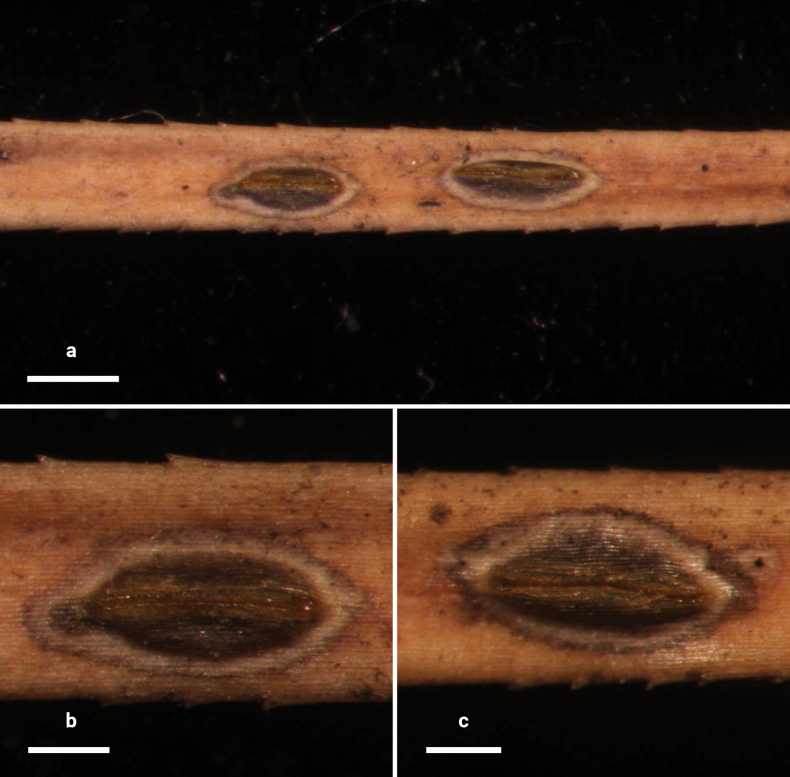
*Lophodermium flavilabioides* on *Pinus koraiensis* (HOU 2230/BJTC 2024090, holotype). **a** Ascomata on needles. **b, c** Mature ascomata and conidiomata. Scale bars: 700 μm (**a**); 300 μm (**b, c**).

**Figure 7.**
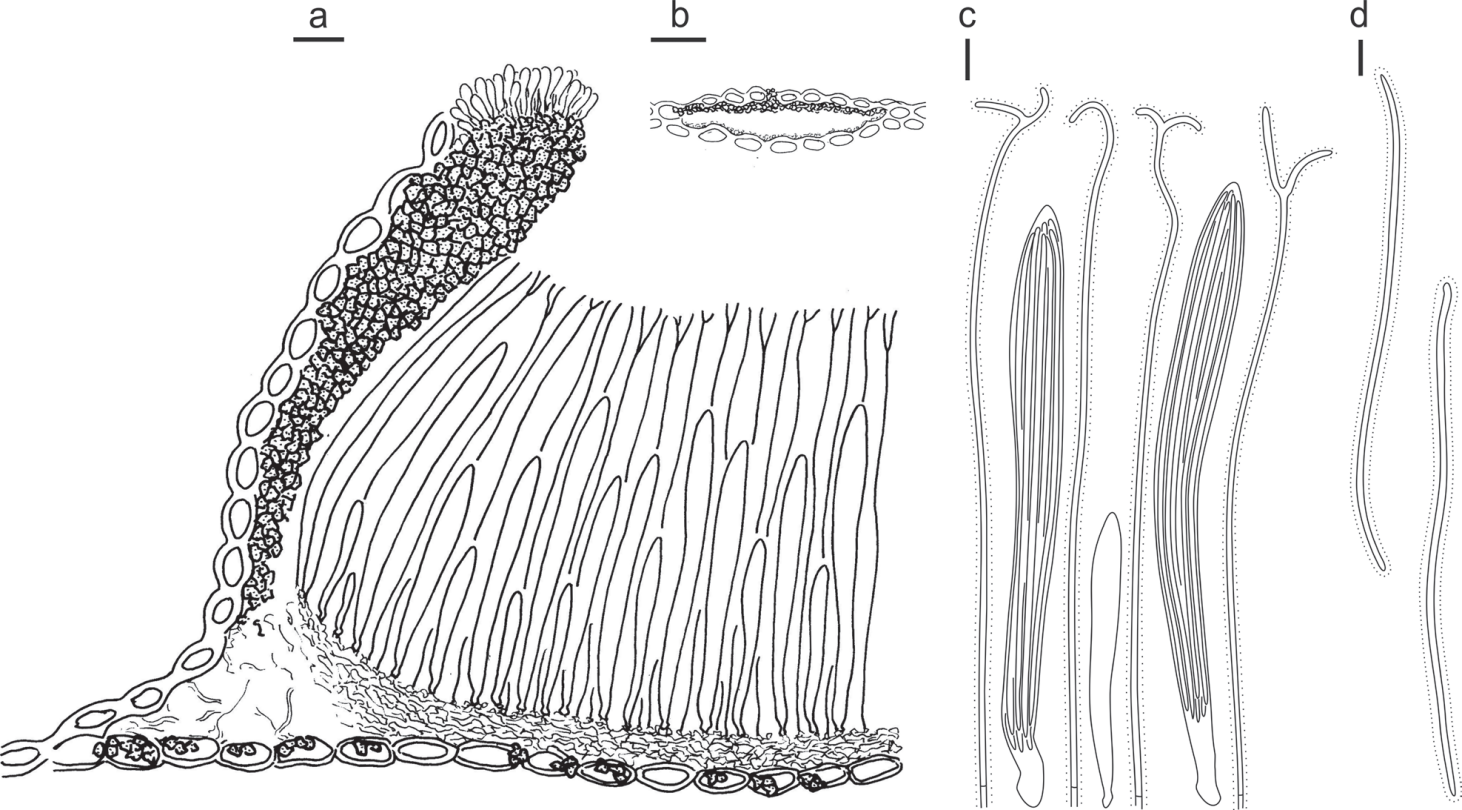
*Lophodermium flavilabioides* on *Pinus koraiensis* (HOU 2230/BJTC 2024090, holotype). **a** Part of an ascoma in vertical section. **b** Conidioma in vertical section. **c** Paraphyses, mature asci with ascospores, and immature ascus. **d** Liberated ascospores. Scale bars: 20 μm (**a**); 50 μm (**b**); 10 μm (**c**); 5 μm (**d**).

##### Type.

CHINA • Heilongjiang Province, Yichun, Wuying National Forest Park, 48°14'16"N, 129°12'18"E, ca. 375 m, on needles of *P. koraiensis*, 16 Jun 2024, *C.L. Hou, L. Zhuo & Y. Gao*, HOU 2230 (**holotype**BJTC 2024090)

##### Etymology.

Referring to the morphological similarity with *Lophodermium flavilabium*.

##### Description.

**Sexual morph: *Ascomata*** mostly on abaxial surface of needles, scattered, associated with pale areas. In surface view, ascomata elliptical, 850–1750 × 450–550 µm, dark gray to black in center with a pale gray border and brown perimeter line, opening by a single longitudinal split. ***Lips*** yellow, embedded in a barely perceptible gelatinous matrix. In median vertical section, ascomata completely subepidermal. ***Covering stroma*** 50–65 μm thick near center of ascomata, not extending to basal stroma, consisting of an outer layer of host cuticle, epidermis, and an inner layer of carbonized, thick-walled angular cells. ***Lip cells*** cylindrical to clavate, 5–15 × 3–5 μm, bright yellow, embedded in a gelatinous matrix. ***Basal stroma*** poorly developed, consisting of carbonized, thick-walled, angular cells. ***Subhymenium*** 10–15 µm thick, consisting of hyaline textura porrecta. ***Paraphyses*** aseptate, filiform, branched at their tips, 140–160 × 1–1.5 µm. ***Asci*** ripening sequentially, cylindrical to clavate, somewhat pointed at the apex, 130–155 × 11–13 µm, thin-walled, J–, 8-spored. ***Ascospores*** aseptate, filiform, 65–130 × ca. 1 μm, hyaline, covered by thin gelatinous sheaths.

**Asexual morph: *Conidiomata*** scattered, round to irregular, 200–300 μm diam., pale gray to concolorous with needle surface, opening by an ostiole. In vertical section, conidiomata subepidermal. ***Upper layer*** 8–15 μm thick, consisting of host cuticle, epidermis, and carbonized angular to globose cells. ***Basal layer*** poorly developed. ***Conidiogenous cells*** and ***conidia*** not seen. ***Zone lines*** not seen.

##### Distribution.

Known only from Heilongjiang Province, China.

##### Notes.

In the phylogenetic tree, sequences of *L. flavilabioides* form a sister clade with *L. flavilabium*. Morphologically, *L. flavilabioides* closely resembles *L. flavilabium*, being nearly indistinguishable in external appearance. However, the paraphyses of *L. flavilabioides* branched at their tips while *L. flavilabium* lacks this feature. Furthermore, the ITS sequence similarity between these two species is 95%. Based on phylogenetic and morphological analyses, *L. flavilabioides* is hereby designated as a new species.

#### Lophodermium haploxyli

Taxon classificationFungiRhytismatalesRhytismataceae

S.J. Wang, L. Zhuo & C.L. Hou sp. nov.

FF6204D0-0E4D-5950-B5E5-6EA020CCCE9B

861655

Figs 8, 9

##### Diagnosis.

This new species can be distinguished from *L. orientale* by larger ascomata and presence of textura prismatica near the opening of covering stroma.

**Figure 8.**
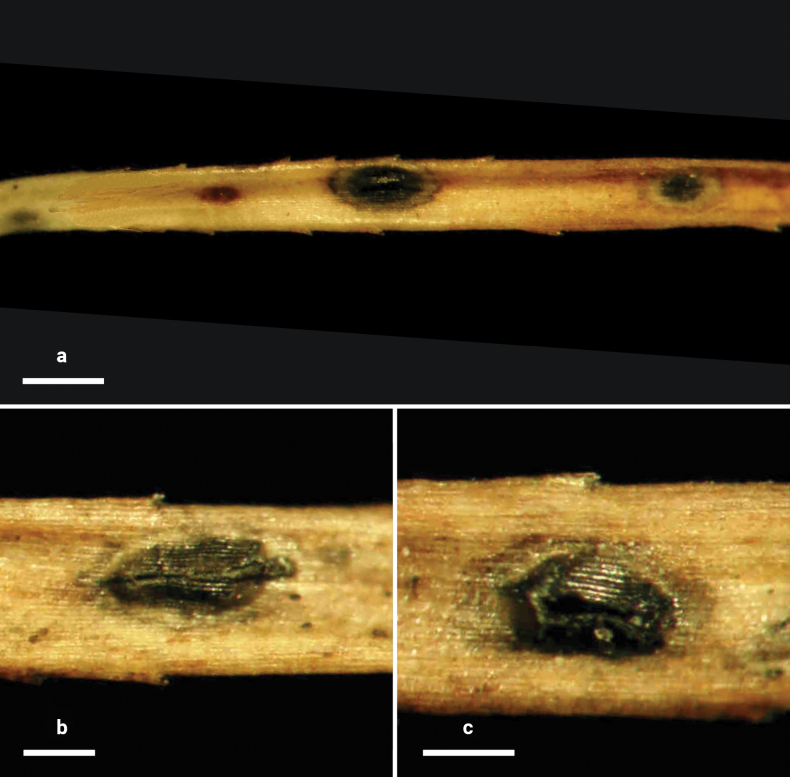
*Lophodermium haploxyli* on *Pinus armandii* (HOU 869A/BJTC 2011031, holotype). **a** Ascomata on needles. **b, c** Mature ascomata. Scale bars: 500 μm (**a**); 250 μm (**b, c**).

**Figure 9.**
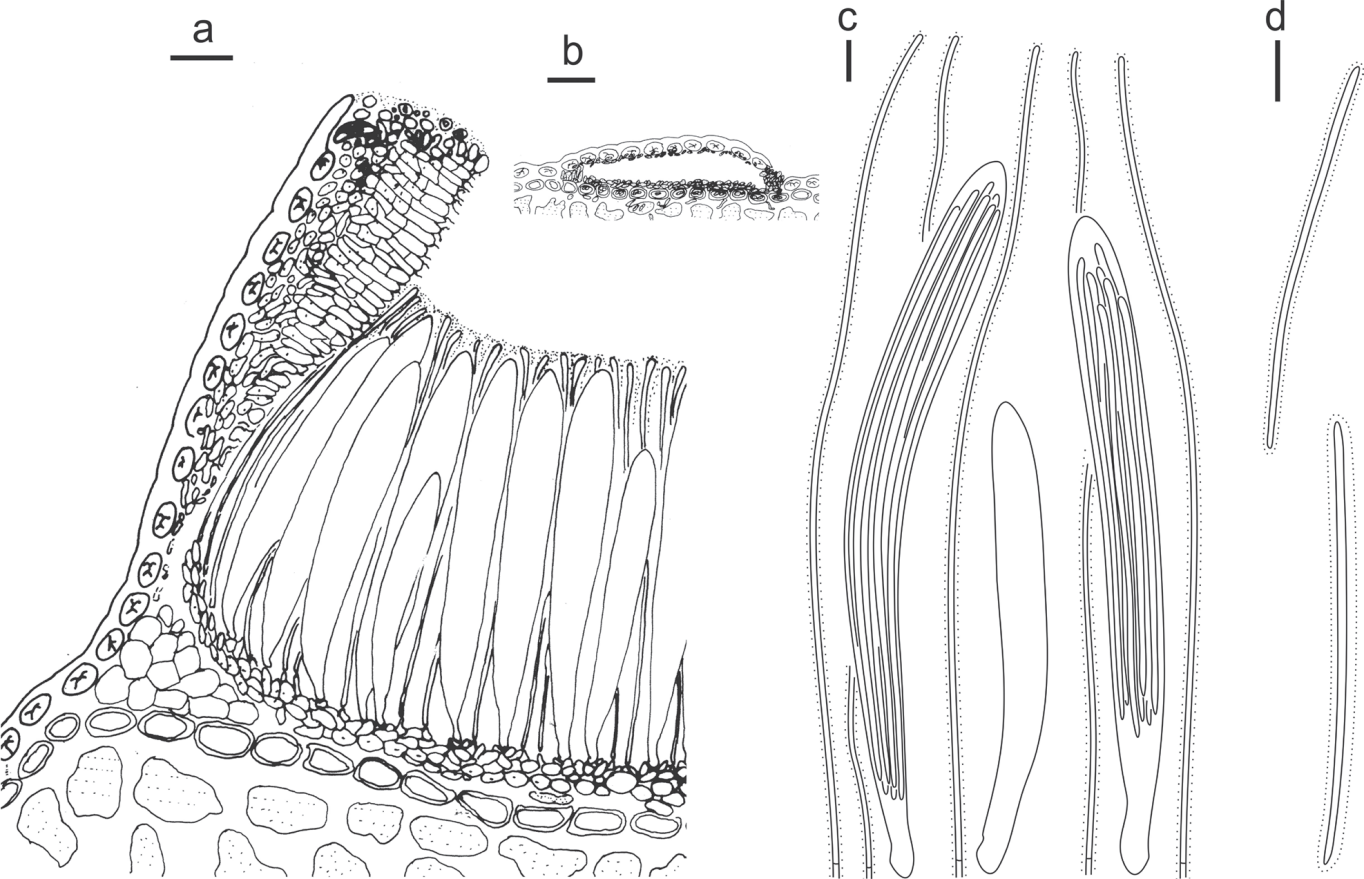
*Lophodermium haploxyli* on *Pinus armandii* (HOU 869A/BJTC 2011031, holotype). **a** Part of an ascoma in vertical section. **b** Conidioma in vertical section. **c** Paraphyses, mature asci with ascospores, and immature ascus. **d** Liberated ascospores. Scale bars: 20 μm (**a**); 50 μm (**b**); 10 μm (**c, d**).

##### Type.

CHINA • Yunnan Province, Lijiang, Laojunshan, on needles of *P. armandii* (*Pinaceae*), 24 June 2011, *C.L. Hou*, HOU 869A (**holotype**BJTC 2011031).

##### Etymology.

From “haploxylon”, referring to the host species are all from *Pinus* subgen. *Strobus*.

##### Description.

**Sexual morph: *Ascomata*** on both sides of needles, scattered, associated with pale areas. In surface view, ascomata elliptical, 420–750 × 300–440 µm, gray to pale grayish black, perimeter line not differentiated, opening by a single longitudinal split. In median vertical section, ascomata subepidermal. ***Covering stroma*** 50–65 μm thick near center of ascomata, thinner towards edges, not extending to basal stroma, consisting of an outer layer of carbonized, thick-walled angular cells underneath epidermal host cells and an inner layer of almost hyaline textura prismatica, with sparse brown melanized fungal elements near opening. ***Basal stroma*** poorly developed. ***Subhymenium*** 8–12 µm thick, consisting of hyaline textura globulosa. A triangular space between covering stroma and basal stroma filled with hyaline large-celled textura angularis. ***Paraphyses*** aseptate or septate, filiform, not branched, not swollen at their tips, 100–120 × ca. 1–1.5 µm. ***Asci*** ripening sequentially, cylindrical, somewhat pointed at apex, 80–110 × 9–12 µm, thin-walled, J–, 8-spored. ***Ascospores*** aseptate, filiform, slightly tapering towards base, 65–90 × 1–1.5 μm, hyaline, covered by thin gelatinous sheath.

**Asexual morph: *Conidiomata*** scattered, elliptical or slightly irregular, 120–400 × 120–260 μm, concolorous with surface of needle or slightly brown, opening by a lateral split. In vertical section, conidiomata subepidermal. ***Upper layer*** consisting of host cuticle, epidermis, and sparse, dark brown fungal tissue. ***Basal layer*** 5–8 μm thick, consisting of carbonized angular to globose cells. ***Conidia*** not seen. ***Zone lines*** black, frequent.

##### Additional specimen examined.

CHINA • Heilongjiang Province, Yichun, Xiaoxing’anling Botanical Garden, 47°44'57"N, 128°53'08"E, ca. 320 m, on needles of *Pinus sibirica* (Ledeb.) Turcz. (*Pinaceae*), 17 Jun 2024, *C.L. Hou, L. Zhuo & Y. Gao*, HOU 2249 (BJTC 2024109); • 47°44'59"N, 128°53'05"E, ca. 300 m, on needles of *P. koraiensis*, 17 Jun 2024, *C.L. Hou, L. Zhuo & Y. Gao*, HOU 2257 (BJTC 2024117).

##### Distribution.

Known from Heilongjiang Province and Yunnan Province, China.

##### Notes.

In the phylogenetic tree, sequences of *L. haploxyli* form a relatively independent clade. Morphologically, *L. haploxyli* is similar to *L. orientale* Minter but differs by the shape of the ascomata, which strongly rise above the substrate, are more pigmented covering the stroma, and include textura prismatica near the opening of the covering stroma (Minter 1981). In addition, sparse brown, granular fungal elements are associated with the opening.

#### Lophodermium huangshanense

Taxon classificationFungiRhytismatalesRhytismataceae

S.J. Wang, L. Zhuo & C.L. Hou sp. nov.

F90939D6-4FB0-51A7-AD93-2EDEE583CC89

861656

Figs 10, 11

##### Diagnosis.

This new species can be distinguished from *L. laojunshanense* by its externally paler and internally darker ascomata, the absence of a hyaline area near the opening of covering stroma, 3–5 epidermal cells being displaced and lying in a group on the basal stroma, and ascospores bearing gelatinous caps. This species can be distinguished from *L. pinastri* by shorter ascomata and ascospores (Darker 1932).

**Figure 10.**
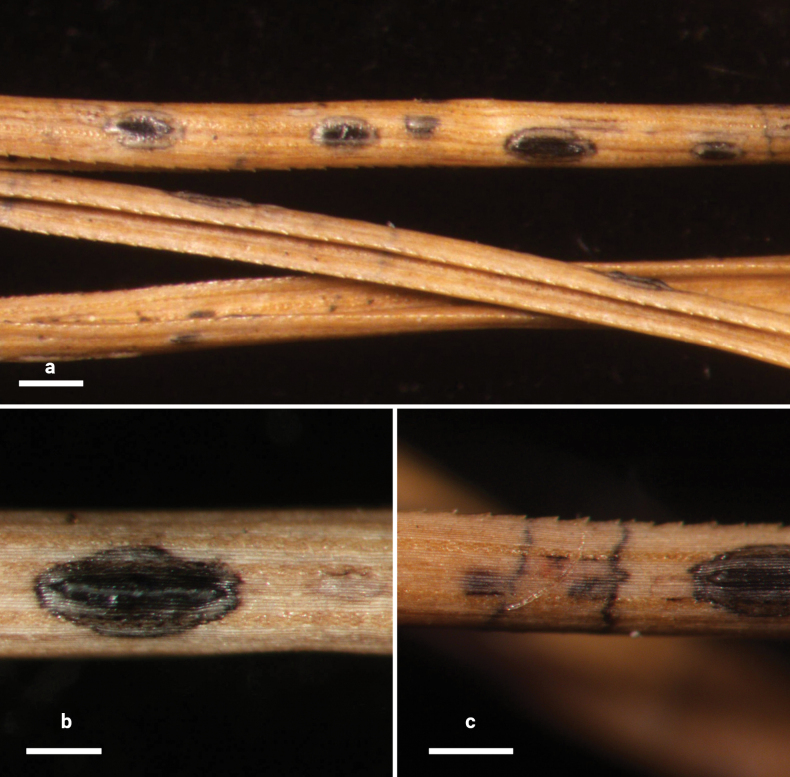
*Lophodermium huangshanense* on *Pinus hwangshanensis* (HOU 1432/BJTC 2019054, holotype). **a** Ascomata on needles. **b, c** Mature ascomata. Scale bars: 500 μm (**a**); 300 μm (**b, c**).

**Figure 11.**
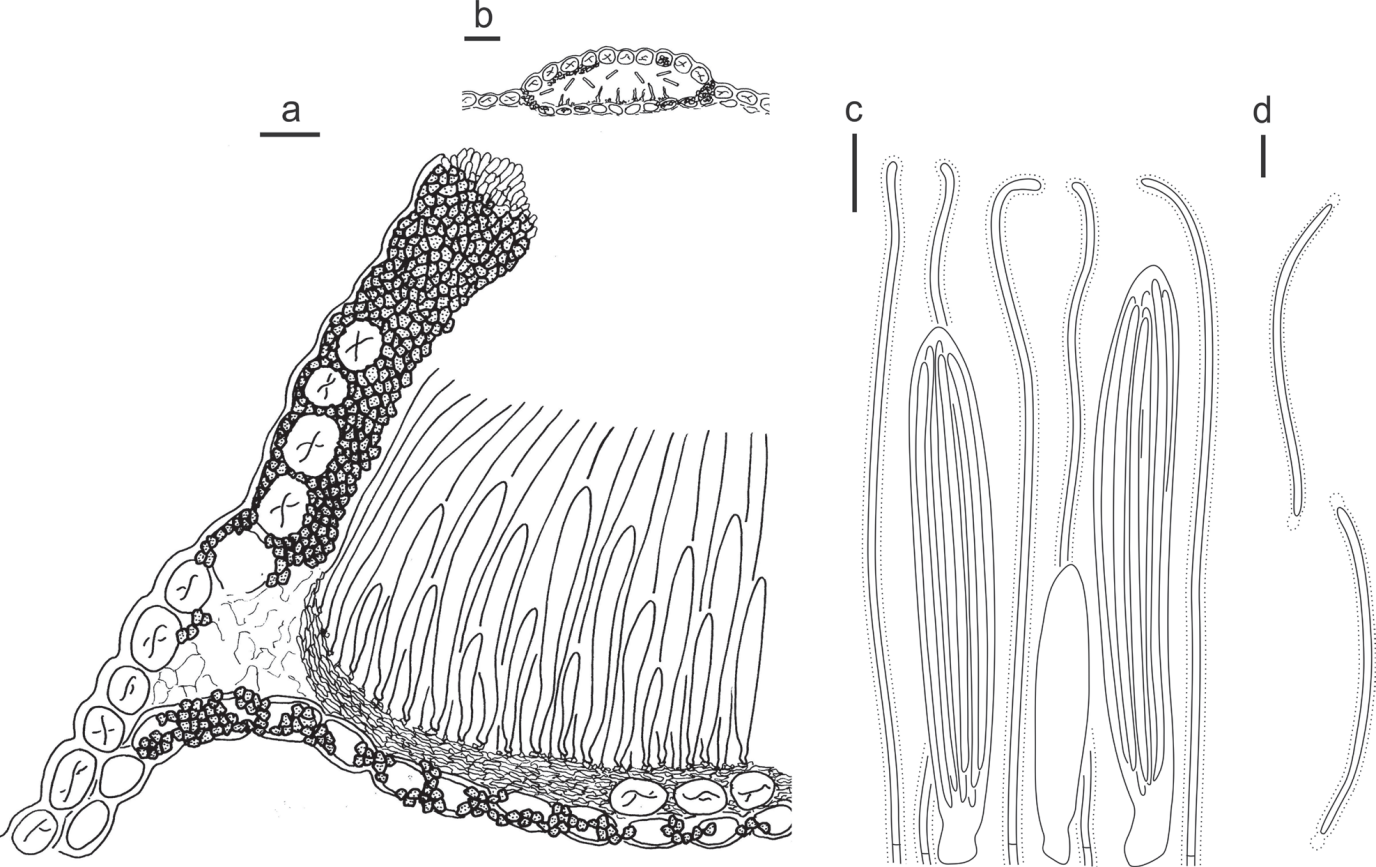
*Lophodermium huangshanense* on *Pinus hwangshanensis* (HOU 1432/BJTC 2019054, holotype). **a** Part of an ascoma in vertical section. **b** Conidioma in vertical section. **c** Paraphyses, mature asci with ascospores, and immature ascus. **d** Liberated ascospores. Scale bars: 20 μm (**a, b**); 10 μm (**c**); 5 μm (**d**).

##### Type.

CHINA • Anhui Province, Huangshan City, Huangshan Mountain Scenic Area, ca. 720 m, on needles of *Pinus hwangshanensis* W.Y. Hsia (*Pinaceae*), 28 Apr 2019, *C.L. Hou & T. Lv*, HOU 1432 (**holotype**BJTC 2019054).

##### Etymology.

Referring to the location (Huangshan) where the specimens were collected.

##### Description.

**Sexual morph: *Ascomata*** mostly on abaxial surface of needles, scattered, associated with pale areas and black perimeter lines. In surface view ascomata elliptical, 700–900 × 300–420 µm, dark gray to black in center with a pale border, black perimeter line present, and opening by a single longitudinal split. In median vertical section, ascomata partly subepidermal. ***Covering stroma*** 30–45 μm thick near center of ascomata, not extending to basal stroma, consisting of an outer layer of host cuticle and an inner layer of carbonized, thick-walled angular cells, that are also present around the epidermal cells. ***Basal stroma*** poorly developed, consisting of carbonized fungal tissue surrounding epidermal cells. ***Subhymenium*** 8–18 µm thick, consisting of hyaline textura porrecta. ***Paraphyses*** aseptate, filiform, not branched, sometimes slightly swollen at their tips, 85–105 × 1 µm. ***Asci*** ripening sequentially, cylindrical, slightly acute at apex, 65–80 × 9–11 µm, thin-walled, J–, 8-spored. ***Ascospores*** aseptate, filiform, 55–70 × ca. 1 μm, hyaline, covered by a thin gelatinous sheath, with gelatinous caps.

**Asexual morph: *Conidiomata*** scattered, elliptical or slightly irregular, 200–300 × 80–100 μm, concolorous or darker than needle surface, opening by lateral splits. In vertical section, conidiomata subepidermal. ***Upper layer*** poorly developed, blackening especially near splits. ***Basal layer*** poorly developed. ***Conidiogenous cells*** cylindrical but tapering towards apex, 5–10 × ca. 1 μm, hyaline. ***Conidia*** cylindrical, 4–7 × 1 μm, hyaline. ***Zone lines*** dark brown to black.

##### Additional specimens examined.

CHINA • Anhui Province, Chizhou, Youhua Forest Farm, on needles of *Pinus* sp., 22 Apr 2024, *C.L. Hou*, HOU 1221 (BJTC 2015007); • Anhui Province, Huangshan, Shexian County, ca. 200 m, on needles of *Pinus massoniana* Lamb. (*Pinaceae*), 14 May 2018, *C.L. Hou, L. Zhuo & X.N. Sui*, HOU 1324 (BJTC 2018020).

##### Distribution.

Known only from Anhui Province, China.

##### Notes.

In the phylogenetic tree, sequences of *L. huangshanense* and *L. laojunshanense* form a clade with a sequence of *L.* cf. *pinastri*. Morphologically, *L. huangshanense* differs from *L. laojunshanense* by its paler externally and darker internally ascomata, the absence of hyaline area near the opening of covering stroma, 3–5 epidermal cells displaced in the covering stroma towards the center, and ascospores bearing gelatinous caps. *Lophodermium huangshanense* is morphologically similar to *L. himalayense* and *L. pinastri*, but differs from *L. himalayense* by having aseptate paraphyses, ascospores with gelatinous caps, and distinct, abundant zone lines (Cannon and Minter 1986). *Lophodermium pinastri* can be distinguished from *L. huangshanense* by its longer ascomata (700–1200 μm) and ascospores (70–110 μm) (Darker 1932).

#### Lophodermium jingpohuense

Taxon classificationFungiRhytismatalesRhytismataceae

L. Zhuo & C.L. Hou sp. nov.

73BF85E7-22CA-57F1-A78D-9860C44D181A

861657

Figs 12, 13

##### Diagnosis.

This new species can be distinguished from *Lophodermium calceolatum* by the presence of lip cells and thinner covering stroma near opening.

**Figure 12.**
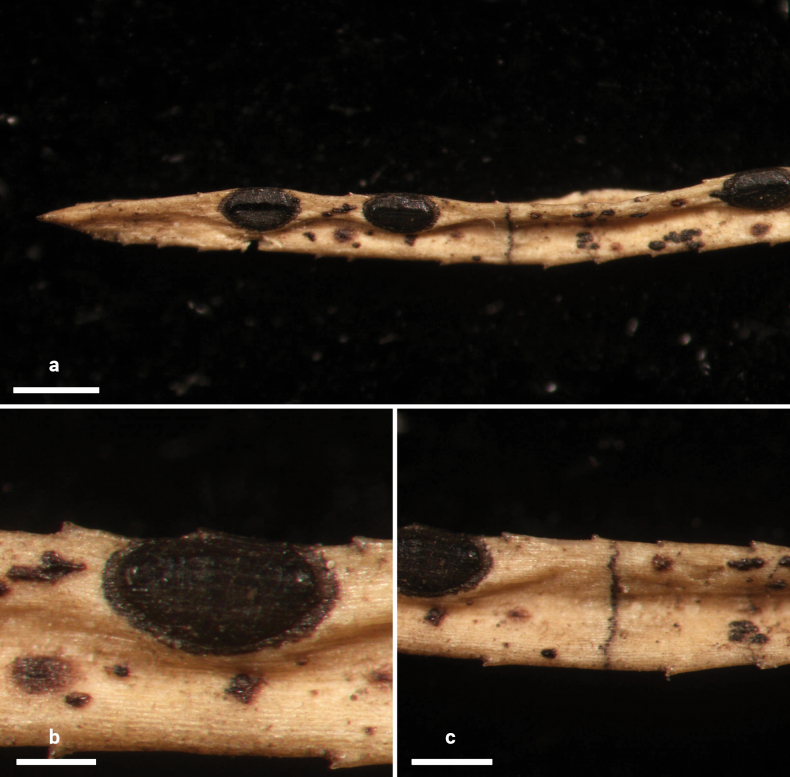
*Lophodermium jingpohuense* on *Pinus koraiensis* (HOU 1100A /BJTC 2013031, holotype). **a** Ascomata on needles. **b** A mature ascoma and conidiomata. **c** Conidiomata and zone line. Scale bars: 1 mm (**a**); 300 μm (**b, c**).

**Figure 13.**
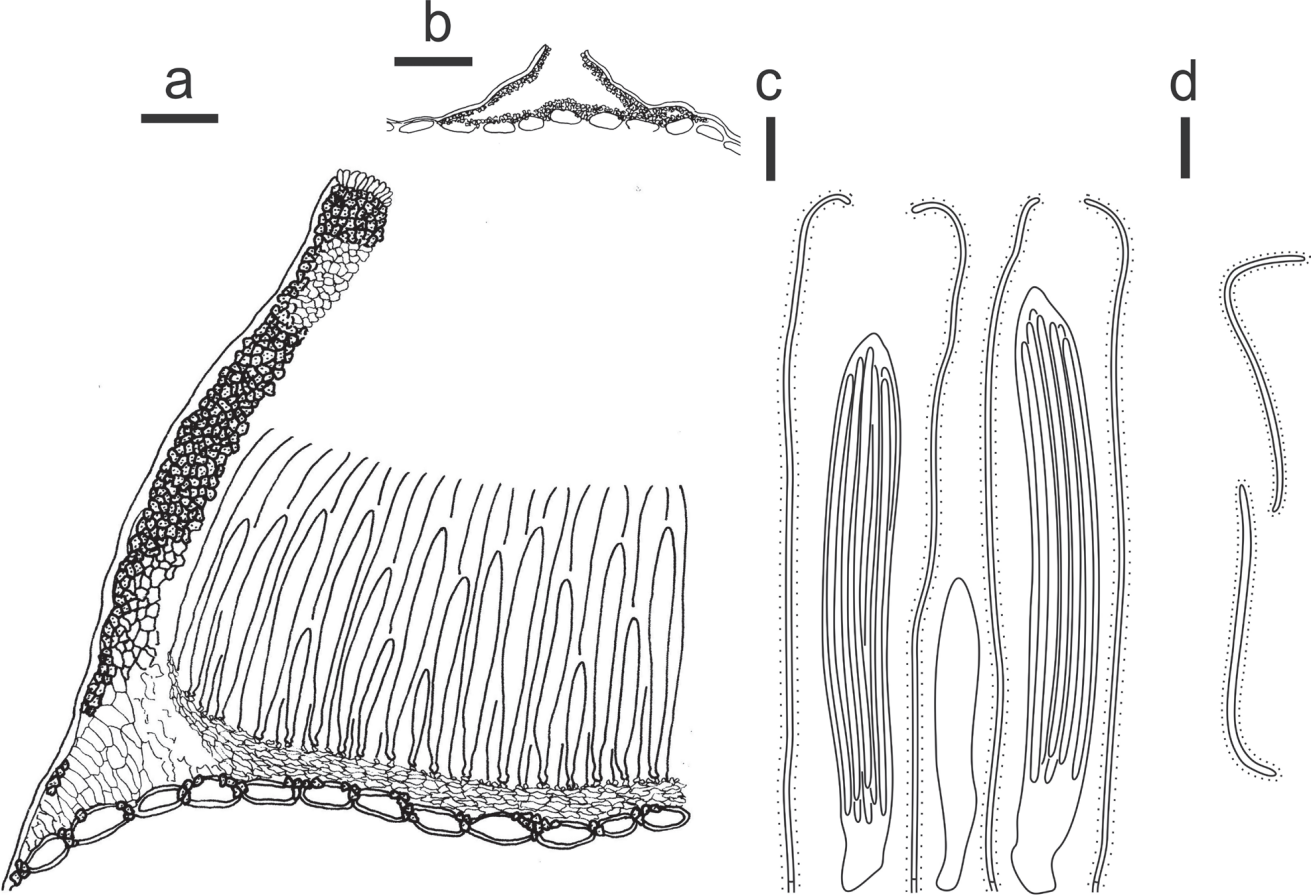
*Lophodermium jingpohuense* on *Pinus koraiensis* (HOU 1100A /BJTC 2013031, holotype). **a** Part of an ascoma in vertical section. **b** Conidioma in vertical section. **c** Paraphyses, mature asci with ascospores, and immature ascus. **d** Liberated ascospores. Scale bars: 30 μm (**a**); 50 μm (**b**); 10 μm (**c, d**).

##### Type.

CHINA • Heilongjiang Province, Ning’an, Jingpohu, on needles of *P. koraiensis*, 17 May 2013, *C.L. Hou*, HOU 1100A (**holotype**BJTC 2013031).

##### Etymology.

Referring to the name of the location (Jingpohu) where the type specimen was collected.

##### Description.

**Sexual morph: *Ascomata*** mostly on abaxial surface of needles, scattered, associated with pale areas. In surface view, ascomata elliptical, 660–870 × 400–500 µm, shiny black with a dark gray to black border and black perimeter line, opening by a single longitudinal split. ***Lips*** inconspicuous. In median vertical section, ascomata subcuticular. ***Covering stroma*** 30–45 μm thick near center of ascomata, extending to basal stroma, consisting of an outer layer of host cuticle and an inner layer of carbonized, thick-walled angular cells. Near opening, with a distinct hyaline zone (30–40 × 10–15 µm) formed by hyaline, thin-walled, angular cells. ***Lip cells*** cylindrical to clavate, 8–12 × 2–3 μm, hyaline. ***Basal stroma*** poorly developed, consisting of carbonized fungal tissue surrounding epidermal cells, in vertical section triangular space between covering stroma and basal stroma at margin of ascoma filled with hyaline textura prismatica. ***Subhymenium*** 20–30 µm thick, consisting of hyaline textura porrecta. ***Paraphyses*** aseptate, filiform, not branched, not swollen at their tips, 80–110 × 1–2 µm. ***Asci*** ripening sequentially, cylindrical to clavate, somewhat pointed at apex, 60–100 × 8–11 µm, thin-walled, J–, 8-spored. ***Ascospores*** aseptate, filiform, 45–70 × 1 μm, hyaline, covered by ca. 2 μm thick gelatinous sheaths.

**Asexual morph: *Conidiomata*** scattered, elliptical to round, 150–300 × 120–180 μm, black, opening by an ostiole. In vertical section, conidiomata subcuticular. ***Upper layer*** 5–7 μm thick, consisting of host cuticle and carbonized angular to globose cells. Basal layer 5–10 μm thick, consisting of carbonized angular to globose cells. ***Conidiogenous cells*** and ***conidia*** not seen. ***Zone lines*** not seen.

##### Additional specimens examined.

CHINA • Heilongjiang Province, Ning’an, Jingpohu, on needles of *P. koraiensis*, 17 May 2013, *C.L. Hou*, HOU 1099A (BJTC 2013030); • Yichun, Wuying National Forest Park, 48°14'17"N, 129°12'119"E, ca. 370 m, on needles of *P. koraiensis*, 24 Jun 2024, *C.L. Hou, L. Zhuo & Y. Gao*, HOU 2231 (BJTC 2024091); • Jilin Province, Yanbian Chaoxianzu Autonomous Prefecture, Changbaishan, 42°31'37"N, 128°16'09"E, ca. 650 m, on needles of *P. koraiensis*, 14 Jun 2024, *C.L. Hou, L. Zhuo & Y. Gao*, HOU 2209 (BJTC 2024069).

##### Distribution.

Known from Jilin Province and Heilongjiang Province, China.

##### Notes.

In the phylogenetic tree, sequences of *L. jingpohuense* form a sister clade with *L. calceolatum*, but the latter lacks lip cells, and its covering stroma is incurved with a right angle at the opening. Morphologically, *L. jingpohuense* closely resembles *Lophodermium nitens*. However, the latter differs by possessing larger ascospores (80–120 × 2–3 µm) and by lacking a hyaline zone of the covering stroma. The sequence similarity of ITS rDNA between *L. nitens* and *L. jingpohuense* is 88%, indicating that *L. jingpohuense* is a distinct species.

#### Lophodermium laojunshanense

Taxon classificationFungiRhytismatalesRhytismataceae

L. Zhuo & C.L. Hou sp. nov.

F4453470-9E6B-5CF6-BBAA-646D8413E9C5

861658

Figs 14, 15

##### Diagnosis.

This new species can be distinguished from *L. huangshanense* by black ascomata, the presence of a hyaline area near the opening of covering stroma, more than 10 epidermal cells displaced in the basal stroma towards the center, and ascospores not bearing gelatinous caps.

**Figure 14.**
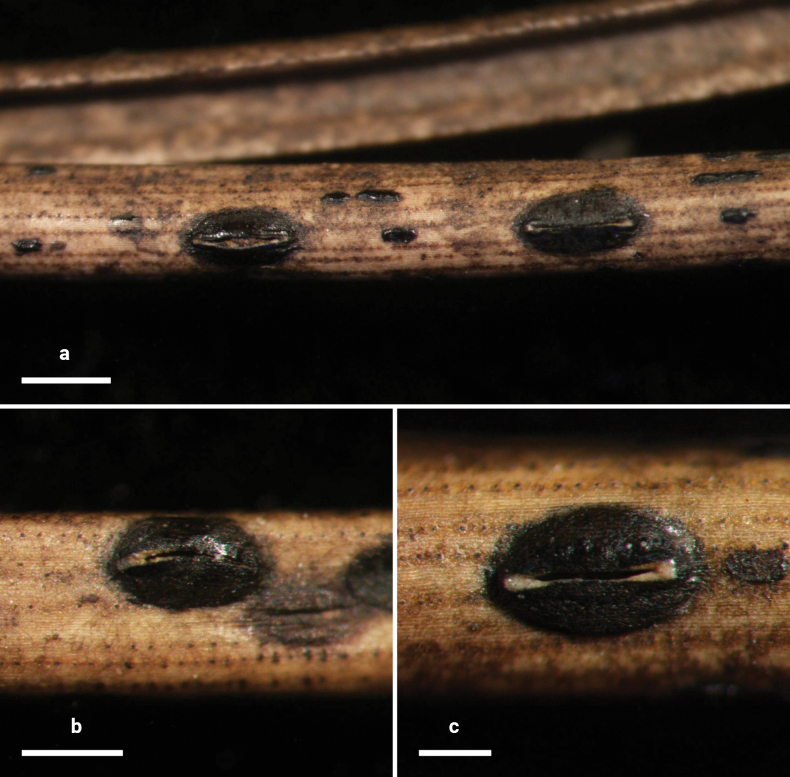
*Lophodermium laojunshanense* on *Pinus yunnanensis* (HOU 2018/BJTC 2023148, holotype). **a** Ascomata on needles. **b, c** Mature ascomata and conidiomata. Scale bars: 1 mm (**a**); 500 μm (**b**); 300 μm (**c**).

**Figure 15.**
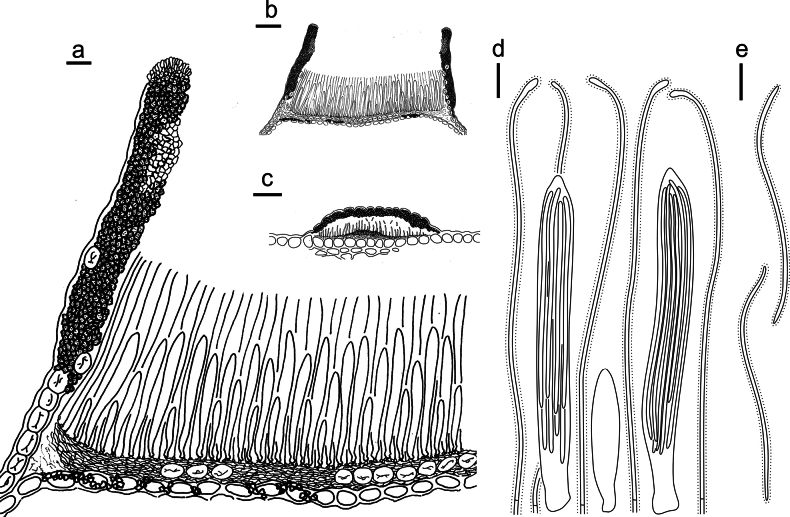
*Lophodermium laojunshanense* on *Pinus yunnanensis* (HOU 2018/BJTC 2023148, holotype). **a** Part of an ascoma in vertical section. **b** An entire ascoma in vertical section. **c** Conidioma in vertical section. **d** Paraphyses, mature asci with ascospores, and immature ascus. **e** Liberated ascospores. Scale bars: 20 μm (**a**); 100 μm (**b**); 50 μm (**c**); 10 μm (**d, e**).

##### Type.

CHINA • Yunnan Province, Lijiang, Laojunshan, 26°39'05"N, 99°46'32"E, ca. 3500 m, on needles of *Pinus yunnanensis* Franch. (*Pinaceae*), 16 Aug 2023, *C.L. Hou, L. Zhuo & S.Y. Zhao*, HOU 2018 (**holotype**BJTC 2023148).

##### Etymology.

Referring to the location (Laojunshan) where the specimens were collected.

##### Description.

**Sexual morph: *Ascomata*** mostly on abaxial surface of needles, scattered, associated with pale areas. In surface view ascomata elliptical, 700–1150 × 400–600 µm, black, shiny, perimeter line not differentiated, opening by a single longitudinal split. ***Lips*** present, white. In median vertical section, ascomata subcuticular to partially subepidermal. ***Covering stroma*** 35–50 μm thick near center of ascomata, not extending to basal stroma, consisting of an outer layer of host cuticle and an inner layer of carbonized, thick-walled angular cells. ***Lip cells*** cylindrical, 4–8 × 2–3 μm, hyaline. ***Basal stroma*** poorly developed. ***Subhymenium*** 8–15 µm thick, consisting of hyaline textura porrecta. ***Paraphyses*** aseptate, filiform, not branched, swollen to 2–3 μm at their tips, 105–125 × ca. 1 µm. ***Asci*** ripening sequentially, cylindrical, somewhat pointed at apex, 65–105 × 10–12 µm, thin-walled, J–, 8-spored. ***Ascospores*** aseptate, filiform, 60–85 × 1 μm, hyaline, covered by thin gelatinous sheaths.

**Asexual morph: *Conidiomata*** scattered, elliptical or slightly irregular, 120–300 × 80–180 μm, black, opening by a lateral split to liberate conidia. In vertical section, conidiomata subcuticular. ***Upper layer*** 5–15 μm thick, consisting of host cuticle and carbonized angular to globose cells. ***Basal layer*** 5–10 μm thick, consisting of host cuticle and carbonized angular to globose cells. ***Conidiogenous cells*** cylindrical but tapering towards apex, 10–20 × ca. 1 μm, hyaline. ***Conidia*** cylindrical, 4–6 × 1 μm, hyaline. ***Zone lines*** not seen.

##### Additional specimen examined.

CHINA • Yunnan Province, Lijiang, Laojunshan, 26°39'59"N, 99°56'20"E, ca. 2345 m, on needles of *P. yunnanensis*, 16 Jul 2020, *C.L. Hou, Q.T. Wang & M.J. Guo*, HOU 1615 (BJTC 2020057).

##### Distribution.

Known only from Yunnan Province, China.

##### Notes.

In the phylogenetic tree, sequences of *L. laojunshanense* form a sister clade to *L. huangshanense*. Morphologically, *L. laojunshanense* differs from *L. huangshanense* by black ascomata, the presence of hyaline area near the opening of the covering stroma, more than ten epidermal cells displaced lying in a group on the basal stroma, and ascospores not bearing gelatinous caps.

#### Lophodermium piceum

Taxon classificationFungiRhytismatalesRhytismataceae

S.J. Wang, L. Zhuo & C.L. Hou sp. nov.

70798641-9A80-50F5-BD23-05B32CEC20B6

861659

Figs 16, 17

##### Diagnosis.

This new species can be distinguished from *L. pini-hwangshanensis* by the poorly developed basal stroma and abundant and dense conidiomata.

**Figure 16.**
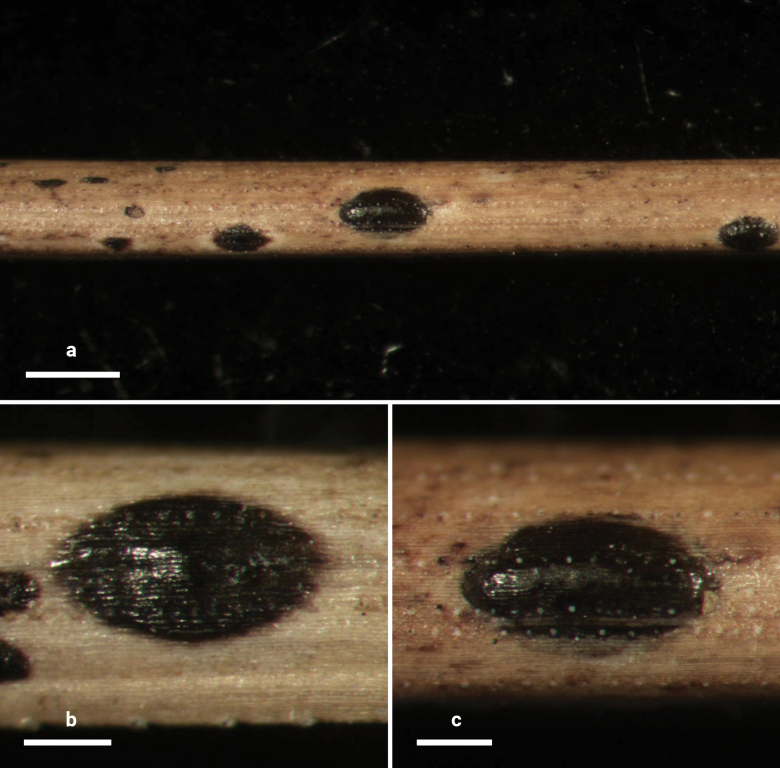
*Lophodermium piceum* on *Pinus densata* (HOU 917A/BJTC 2011075, holotype). **a** Ascomata on needles. **b, c** Mature ascomata. Scale bars: 1 mm (**a**); 300 μm (**b, c**).

**Figure 17.**
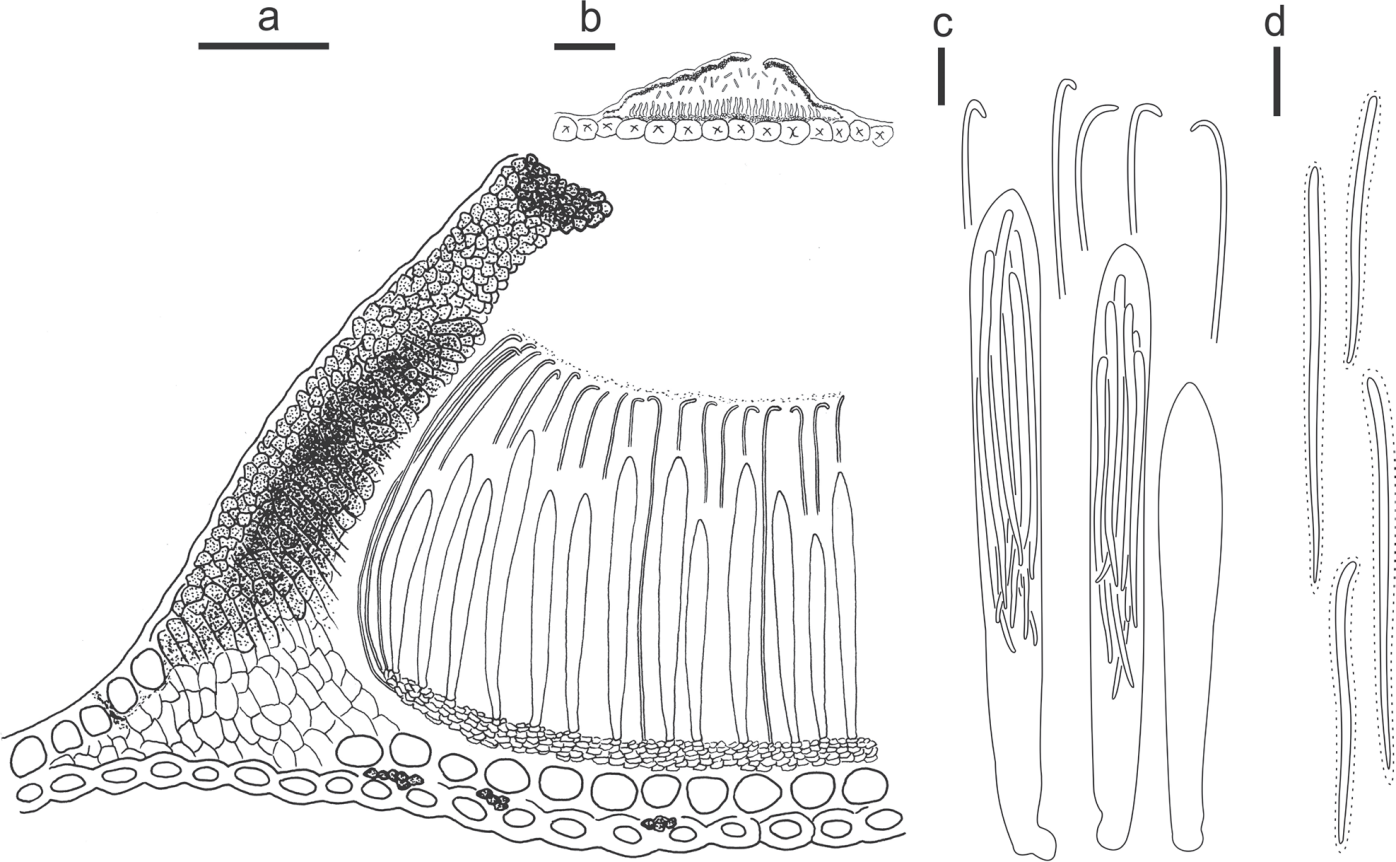
*Lophodermium piceum* on *Pinus densata* (HOU 917A/BJTC 2011075, holotype). **a** Part of an ascoma in vertical section. **b** Conidioma in vertical section. **c** Paraphyses, mature asci with ascospores, and immature ascus. **d** Liberated ascospores. Scale bars: 50 μm (**a, b**); 10 μm (**c, d**).

##### Type.

CHINA • Yunnan Province, Diqing Zangzu Autonomous Prefecture, Shangri-la, ca. 3200 m, on needles of *Pinus densata* Mast. (*Pinaceae*), 27 Jun 2011, *C.L. Hou*, HOU 917A (**holotype**BJTC 2011075).

##### Etymology.

*piceus* (Latin) = pitch black, referring to the color of the ascomata.

##### Description.

**Sexual morph: *Ascomata*** mostly on abaxial surface of needles, scattered, associated with pale areas. In surface view ascomata elliptical, 660–1000 × 325–500 µm, black, shiny, perimeter line not differentiated, opening by a single longitudinal split. In median vertical section, ascomata subcuticular to partly subepidermal. ***Covering stroma*** 40–55 μm thick near center of ascomata, recurved inward at opening, not extending to basal stroma, consisting of an outer layer of host cuticle, an inner layer of carbonized, thick-walled angular cells and an innermost layer of textura prismatica. ***Basal stroma*** poorly developed. ***Subhymenium*** 13–20 µm thick, consisting of hyaline textura porrecta. A triangular space between covering stroma and basal stroma filled with hyaline large-celled textura angularis and textura prismatica. ***Paraphyses*** aseptate or septate, filiform, not branched, not swollen at their tips, 110–125 × ca. 1 µm. ***Asci*** ripening sequentially, cylindrical, somewhat pointed at apex, 65–120 × 10–14 µm, thin-walled, J–, 8-spored. ***Ascospores*** aseptate, filiform, slightly tapering towards base, 45–105 × 1–1.5 μm, hyaline, covered by thin gelatinous sheaths.

**Asexual morph: *Conidiomata*** abundant, scattered, elliptical or slightly irregular, 100–400 × 75–175 μm, brown to black, opening by a lateral or irregular split. In vertical section, conidiomata subcuticular. ***Upper layer*** 4–5 μm thick, consisting of host cuticle and carbonized angular to globose cells. ***Basal layer*** absent. ***Conidia*** cylindrical, 7–9 × 0.6–0.8 μm, hyaline. ***Zone lines*** grayish-brown, slender.

##### Additional specimens examined.

CHINA • Hebei Province, Chengde, Wulingshan, on needles of *Pinus tabuliformis* Carrière (*Pinaceae*), 28 Aug 2010, *C.L. Hou*, HOU 812 (BJTC 2010011); • Gansu Province, Gannan Tibetan Autonomous Prefecture, Diebu County, 34°00'01"N, 103°24'09"E, ca. 2270 m, on needles of *Pinus* sp., 09 Aug 2023, *C.L. Hou, M.J. Guo & X.N. Sui*, HOU 1948 (BJTC 2023078); • Shanxi Province, Yuncheng, Shunwangping, 35°25'47"N, 111°57'54"E, ca. 2230 m, on needles of *P. tabuliformis*, 04 Aug 2024, *C.L. Hou, L. Zhuo & X.N. Sui*, HOU 2299 (BJTC 2024149).

##### Distribution.

Known from Hebei Province, Gansu Province and Shanxi Province, China.

##### Notes.

The multi-locus phylogenetic analysis indicates that the sequences of the specimen of *Lophodermium piceum* form a well-supported clade sister to the sequences of *L. pini-hwangshanensis*. *Lophodermium pini-hwangshanensis*, however, has a well-developed basal stroma, distinct hyaline area near the opening of the covering stroma, and gray conidiomata with lateral splits. Furthermore, the sequence similarity of ITS rDNA between *L. piceum* and *L. pini-hwangshanensis* is only 94%. Therefore, based on morphological characteristics and molecular data, these new specimens are presented as new species.

#### Lophodermium pini-hwangshanensis

Taxon classificationFungiRhytismatalesRhytismataceae

S.J. Wang, L. Zhuo & C.L. Hou sp. nov.

2D7946D2-E19E-5EBF-A5C3-341F6BB87E78

861660

Figs 18, 19

##### Diagnosis.

This new species can be distinguished from *L. piceum* by the presence of a basal stroma.

**Figure 18.**
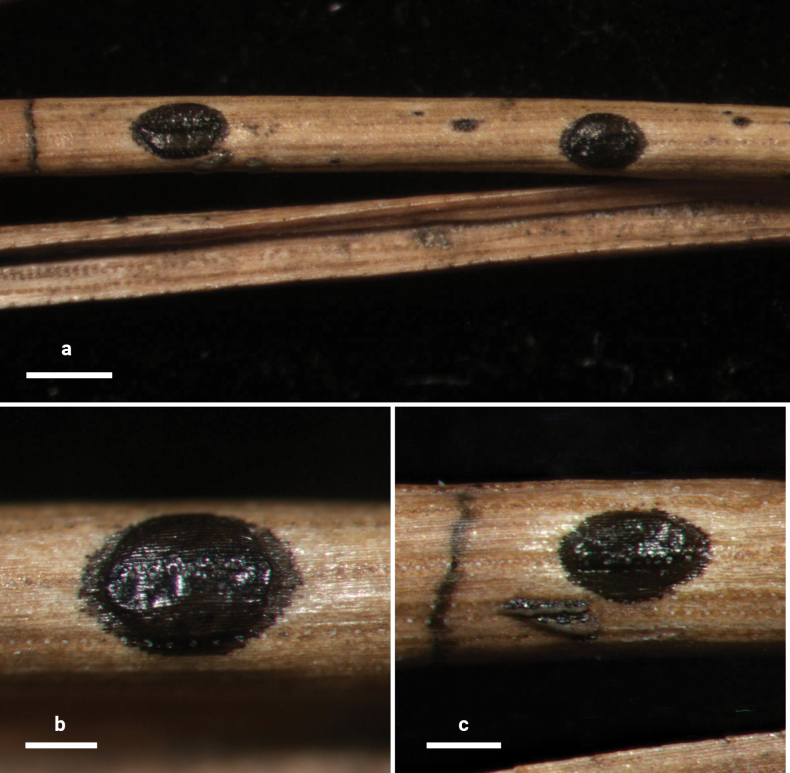
*Lophodermium pini-hwangshanensis* on *Pinus hwangshanensis* (HOU 1092A/BJTC 2013023, holotype). **a** Ascomata on needles. **b** A mature ascoma. **c** An ascoma, conidiomata and zone line. Scale bars: 800 μm (**a**); 300 μm (**b, c**).

**Figure 19.**
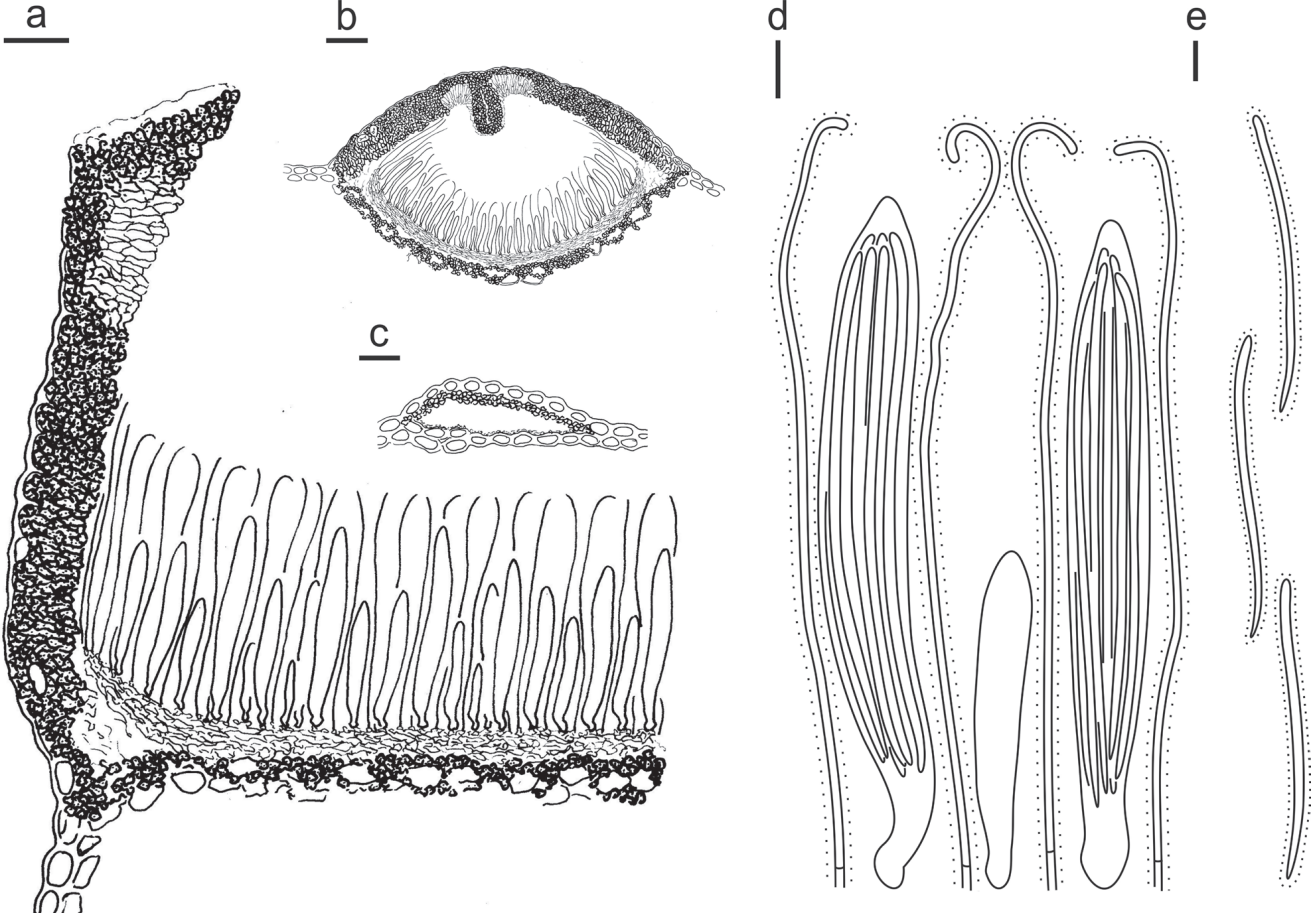
*Lophodermium pini-hwangshanensis* on *Pinus hwangshanensis* (HOU 1092A/BJTC 2013023, holotype). **a** Part of an ascoma in vertical section. **b** An entire ascoma in vertical section. **c** Conidioma in vertical section. **d** Paraphyses, mature asci with ascospores, and immature ascus. **e** Liberated ascospores. Scale bars: 30 μm (**a**); 50 μm (**b, c**); 10 μm (**d, e**).

##### Type.

CHINA • Anhui Province, Huangshan, Huangshan Mountain Scenic Area, on needles of *P. hwangshanensis*, 13 May 2013, *C.L. Hou*, HOU 1092A (**holotype**BJTC 2013023).

##### Etymology.

Referring to the host, *P. hwangshanensis*.

##### Description.

**Sexual morph: *Ascomata*** mostly on abaxial surface of needles, scattered, associated with pale areas. In surface view ascomata elliptical, 600–800 × 400–500 µm, black, shiny, perimeter line not differentiated, opening by a single longitudinal split. In median vertical section, ascomata subcuticular to partly subepidermal. ***Covering stroma*** 40–50 μm thick near center of ascomata, recurved inward at opening, extending to basal stroma, consisting of an outer layer of host cuticle, an inner layer of carbonized, thick-walled angular cells; near opening, a distinct hyaline zone of covering stroma formed by textura prismatica; a melanized textura prismatica structure of covering stroma also present near base. ***Basal stroma*** 10–20 μm thick, consisting of carbonized, thick-walled angular cells. ***Paraphyses*** aseptate, filiform, not branched, not swollen at their tips, with guttulate bases, 100–130 × 1–2 µm. ***Asci*** ripening sequentially, cylindrical, somewhat pointed at apex, 60–95 × 10–12 µm, thin-walled, J–, 8-spored. ***Ascospores*** aseptate, filiform, slightly tapering towards base, 60–80 × ca. 1 μm, hyaline, covered by thin gelatinous sheath.

**Asexual morph: *Conidiomata*** scattered, elliptical or slightly irregular, 200–400 × 80–120 μm, gray, opening by a lateral or irregular split. In vertical section, conidiomata subepidermal. ***Upper layer*** 5–10 μm thick, consisting of host cuticle and carbonized angular to globose cells. ***Basal layer*** absent. ***Conidia*** not seen. ***Zone lines*** gray to black.

##### Other specimen examined.

CHINA • Anhui Province, Anqing, Yuexi, on needles of *P. hwangshanensis*, 10 Jul 2024, *C.L. Hou*, HOU 514 (BJTC 2007078); • Ibid., 20 Apr 2015, *C.L. Hou*, HOU 1220 (BJTC 2015006); • Huangshan, Huangshan Mountain Scenic Area, 30°08'28"N, 118°10'15"E, ca. 1640 m, on needles of *P. hwangshanensis*, 24 Jun 2024, *M.J. Guo*, HOU 1919 (BJTC 2023048).

##### Distribution.

Known only from Anhui Province, China.

##### Notes.

Phylogenetic analyses show that *L. pini-hwangshanensis* and *L. piceum* together form a strongly supported clade. However, they differ from each other because the latter has a poorly developed basal layer in the ascomata, a less distinct hyaline area near the opening of the covering stroma, and abundant, dense conidiomata. Furthermore, the sequence similarity of ITS rDNA between *L. pini-hwangshanensis* and *L. piceum* is only 94%. Therefore, based on morphological characteristics and molecular data, these new specimens are presented as new species.

#### Lophodermium plumbeum

Taxon classificationFungiRhytismatalesRhytismataceae

L. Zhuo & C.L. Hou sp. nov.

632FAD3D-A0CB-5097-A84C-D0D330E2EEEE

861661

Figs 20, 21

##### Diagnosis.

This new species can be distinguished from *Lophodermium yuexiense* by paraphyses with spirals at their tips.

**Figure 20.**
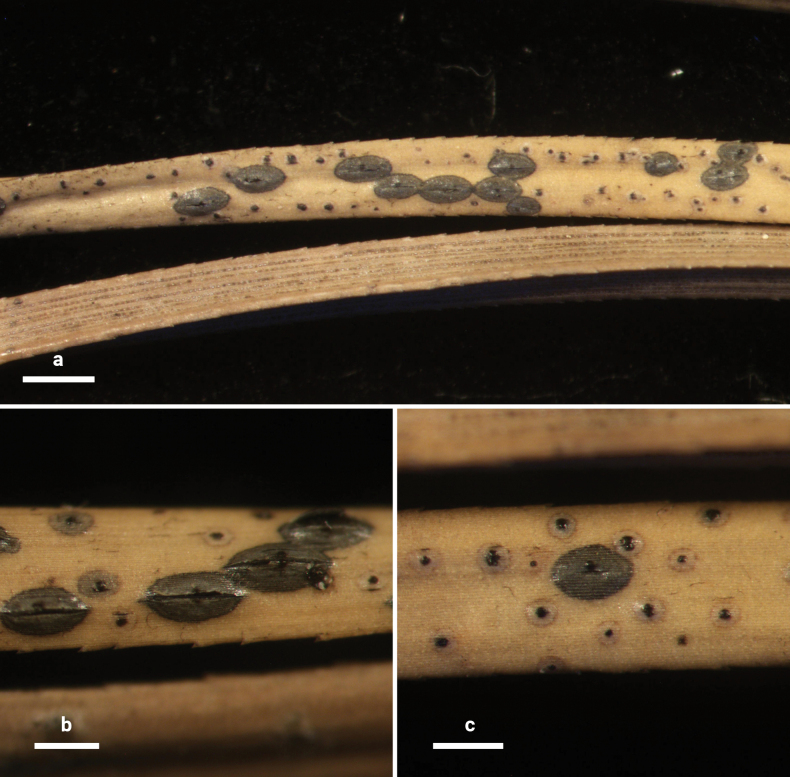
*Lophodermium plumbeum* on *Pinus koraiensis* (HOU 2234/BJTC 2024094, holotype). **a** Ascomata on needles. **b** Mature ascomata. **c** Ascoma and conidiomata. Scale bars: 1 mm (**a**); 500 μm (**b, c**).

**Figure 21.**
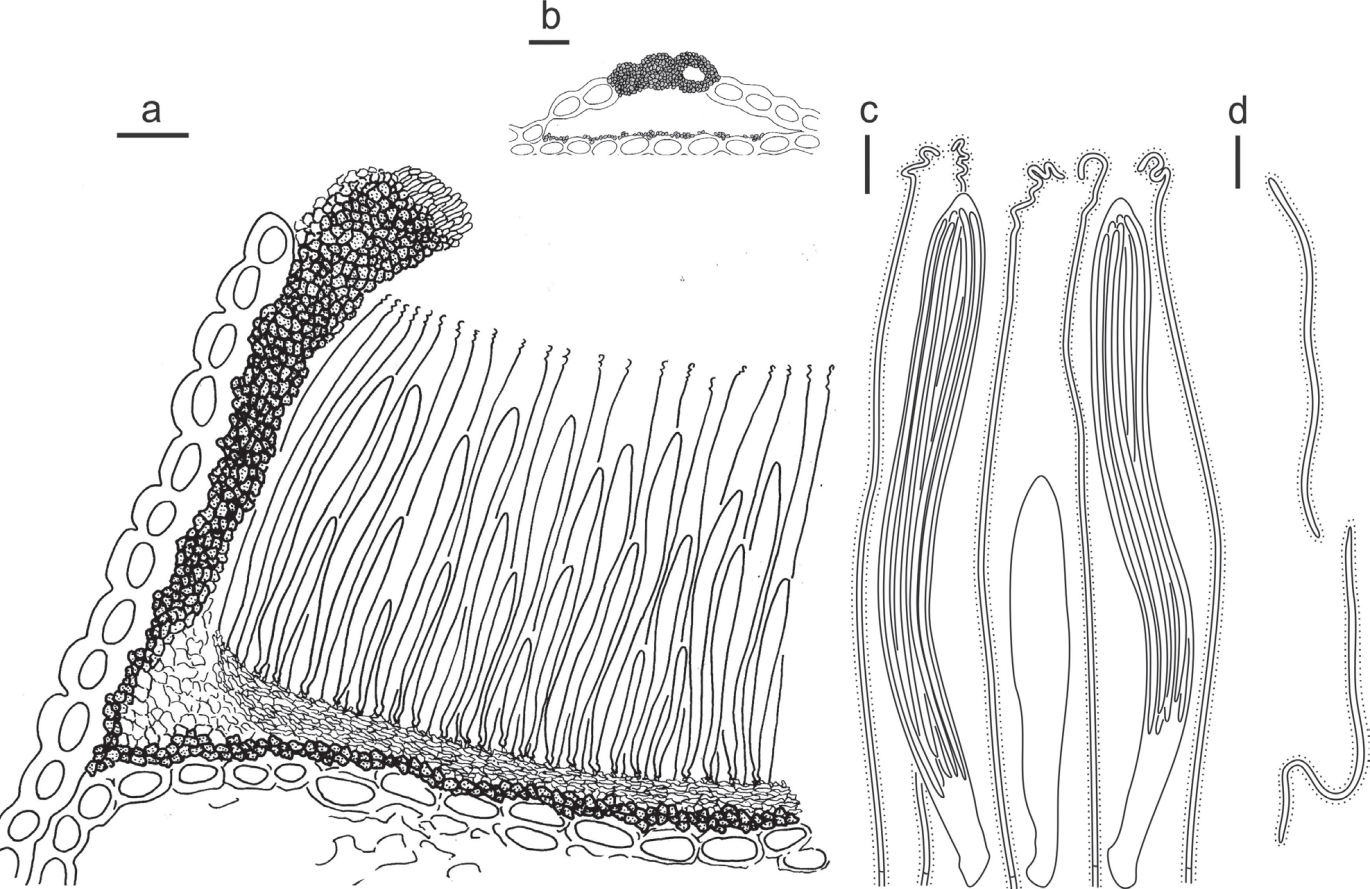
*Lophodermium plumbeum* on *Pinus koraiensis* (HOU 2234/BJTC 2024094, holotype). **a** Part of an ascoma in vertical section. **b** Conidioma in vertical section. **c** Paraphyses, mature asci with ascospores, and immature ascus. **d** Liberated ascospores. Scale bars: 25 μm (**a**); 20 μm (**b**); 10 μm (**c, d**).

##### Type.

CHINA • Heilongjiang Province, Yichun, Wuying National Forest Park, 48°15'10"N, 129°12'10"E, ca. 445 m, on needles of *P. koraiensis*, 16 Jun 2024, *C.L. Hou, L. Zhuo & Y. Gao*, HOU 2234 (**holotype**BJTC 2024094).

##### Etymology.

*plumbeum* (Latin) = leaden, referring to the macroscopical color of the ascomata.

##### Description.

**Sexual morph: *Ascomata*** mostly on abaxial surface of needles, scattered, occasionally coalescing, associated with pale areas. In surface view ascomata elliptical, 600–800 × 300–450 µm, dark gray with darker perimeter line, opening by a single longitudinal split. ***Lips*** inconspicuous. In median vertical section, ascomata subepidermal. ***Covering stroma*** 30–45 μm thick near center of ascomata, thinner towards edges, extending to basal stroma, consisting of an outer layer of host cuticle, host epidermis, and an inner layer of carbonized, thick-walled angular cells. ***Lip cells*** cylindrical, 6–10 × 2–3 μm, hyaline. ***Basal stroma*** 10–20 µm thick, consisting of carbonized, thick-walled, angular cells. ***Subhymenium*** 10–15 µm thick, consisting of hyaline textura porrecta. ***Paraphyses*** aseptate, filiform, not branched, spiral at their tips, 130–150 × 1 µm. ***Asci*** ripening sequentially, cylindrical to clavate, somewhat pointed at apex, 85–140 × 8–10 µm, thin-walled, J–, 8-spored. ***Ascospores*** aseptate, filiform, 60–80 × 1 μm, hyaline, covered by gelatinous sheath 1–2 μm thick.

**Asexual morph: *Conidiomata*** scattered, round, 120–280 μm diam., gray or concolorous with needle surface, surrounded by a dark perimeter line, opening by an ostiole with black periphery. In vertical section, conidiomata partly subcuticular. ***Upper layer*** present only around the ostiole, 15–20 μm thick, consisting of host cuticle and carbonized angular to globose cells. ***Basal layer*** poorly developed. ***Conidiogenous cells*** and ***conidia*** not seen. ***Zone lines*** not seen.

##### Additional specimen examined.

CHINA • Heilongjiang Province, Yichun, Xiao Xing’anling Botanical Garden, 47°44'57"N, 128°53'08"E, ca. 320 m, on needles of *P. sibirica*, 17 Jun 2024, *C.L. Hou, L. Zhuo & Y. Gao*, HOU 2246C (BJTC 2024106).

##### Distribution.

Known only from Heilongjiang Province, China.

##### Notes.

On the phylogenetic tree, sequences of *Lophodermium plumbeum* form a sister clade with *Lophodermium* cf. *pinastri* (Lantz and Williams 417). This species was described in Lantz et al. (2011) as resembling *Lophodermium pinastri*, but differing morphologically by having only three epidermal cells displaced on the basal wall instead of the usual five or more. *Lophodermium plumbeum* is morphologically distinct from *L. pinastri* by gray ascomata and spiral paraphyses tips, confirming that they represent separate species. Morphologically, *L. plumbeum* closely resembles *L. yuexiense*; however, *L. yuexiense* exhibits non-spiral tips of paraphyses and septate ascospores. The sequence similarity of ITS rDNA between *L. yuexiense* and *L. plumbeum* is 84%, indicating that *L. plumbeum* is a distinct species.

## Discussion

### Generic boundaries of *Lophodermium*

The genus *Lophodermium* is typified by *Lophodermium arundinaceum* (Schrad.) Chevall., a species occurring on monocotyledonous hosts. Previous studies have demonstrated that some species of *Rhytismatales* exhibit strong host specificity (Wang et al. 2014; Wang et al. 2023; Guo et al. 2024). Phylogenetic analyses in the present study, as well as prior work (Ortiz García et al. 2003; Lantz et al. 2011), confirm that species of *Lophodermium* closely related to the type (*L. arundinaceum*) are exclusively associated with monocotyledonous hosts, forming a well-supported clade of *Lophodermium* sensu stricto (clade 2). These findings confirm that the genus *Lophodermium* needs to be redefined to include only taxa associated with herbaceous plants (Lantz et al. 2011). However, this hypothesis currently lacks robust phylogenetic support, due to the considerable size of the genus and the scarcity of molecular data for many of its species. Intensive collection and investigation of *Lophodermium* is required on a global scale.

Although the newly described species in this study align with the traditional morphological concept of *Lophodermium*, they do not cluster with its type species, *L. arundinaceum*. Rather, they are scattered across two distinct clades. The majority of *Lophodermium* species associated with *Pinus* needles belongs to a large clade (clade 1). The type species of *Elytroderma* and *Meloderma*, as well as some species associated with *Pinaceae*, such as *Ploioderma pini-armandii* and *Soleella pinicola* form part of this clade. These species develop ascospores with diverse shapes (bifusiform, filiform, fusiform-cylindrical) indicating that the shape of ascospores is unsuitable for genus differentiation (Lantz et al. 2011). Therefore, generic delimitation should be based on an integrative assessment of multiple traits, including host, ascomata morphology, and other relevant morphological features.

In addition to the major clades, a newly discovered species, *Lophodermium haploxyli*, along with other *Lophodermium* species from *Pinus* and taxa from *Abies* (e.g., *Lophodermium piceae*) and *Picea* (e.g., *Lirula macrospora*), form a distinct, separate branch (clade 3) in the phylogenetic tree. While the polyphyly of *Lophodermium* is evident, creating separate genera for each clade would result in excessive splitting, particularly given the unresolved relationships within clade 1. We therefore adopt a conservative approach, retaining all newly described species in *Lophodermium* s.l. pending broader taxon sampling (e.g., inclusion of unsampled Asian and South American taxa) and multi-locus phylogenomic analyses.

### Species similar to *Lophodermium kumaunicum*

The two new species described in this study, *Lophodermium piceum* and *Lophodermium pini-hwangshanensis*, are morphologically similar to *L. kumaunicum* as originally described (Minter and Sharma 1982). However, the phylogenetic placement of *L. kumaunicum* remains unclear due to the lack of molecular data derived from its type specimen. None of the ITS sequences currently deposited in GenBank under the name *L. kumaunicum* are derived from the type specimen or a verified type culture, and therefore their taxonomic identity cannot be considered authoritative for defining the species concept of *L. kumaunicum*.

Sequences from NCBI previously identified as *L. kumaunicum* are also included in present phylogenetic analyses (clade 1). Based on sequence similarity and phylogenetic placement, specimen 514A is reidentified as *L. pini-hwangshanensis* and specimen 812A as *L. piceum*. These reidentifications indicate that at least part of the sequence data currently attributed to *L. kumaunicum* in public databases represent other species within *Lophodermium* rather than authentic *L. kumaunicum*.

Two additional culture-derived sequences, isolates 21 and 22 (accession number EU696776 and EU696777), obtained from needles of *Pinus densata*, share 88% ITS sequence similarity with the sequences of *L. piceum* and *L. pini-hwangshanensis*, indicating that they are clearly distinct from the two new species. In phylogenetic analyses, these isolates cluster with *Lophodermium cathayae* and share 98% ITS similarity to *L. cathayae*. However, since *L. cathayae* differs from *L. kumaunicum* by many morphological traits (larger ascomata, different in color, wider asci, and subepidermal conidiomata), the taxonomic identity of these isolates cannot be confirmed at present and requires additional specimen collection and examination. Additional specimens of *L. kumaunicum*, ideally including material suitable for molecular analysis, are needed to clarify the phylogenetic position and species concept of this taxon.

Morphologically, although similar, there are also some differences between *L. kumaunicum* and the two new species. The ascomata of *L. piceum* and *L. pini-hwangshanensis* are distinctly broader and more rounded at both ends compared to *L. kumaunicum*. Additionally, *L. piceum* differs from *L. kumaunicum* in possessing covering stroma and triangular zone composed of textura prismatica, lacking lips, and exhibiting paraphyses with non-swollen tips. Similarly, *L. pini-hwangshanensis* can be distinguished from *L. kumaunicum* by its hyaline area at the covering stroma opening composed of textura prismatica, a thicker basal stroma, and paraphyses with non-swollen tips (Minter and Sharma 1982).

### Host specificity

Host specificity is important to species delimitation in *Rhytismatales*. Previous work has shown that host association, when evaluated together with morphological characters and molecular phylogenetic evidence, can provide a useful delimitation of species boundaries. For example, Wang et al. (2024) proposed *Rhytismataceae* s.s. based on a combination of morphology, phylogeny, and host specificity. Similar patterns of host-related phylogenetic structure have been reported in other groups of *Rhytismatales*, closely related fungal species tend to occur on closely related host lineages (Guo et al. 2024, 2025).

In the present study, all newly described species were collected from needles of *Pinus*, and each species was restricted to one or several host species belonging to the same *Pinus* subsection. Phylogenetic analyses suggest a preliminary correspondence between host association and species delimitation, species occurring on the same host lineage tend to cluster together phylogenetically. For example, *Lophodermium jingpohuense*, *L. calceolatum*, and *L. fissuratum*, which form a monophyletic group in the phylogenetic analyses and are all associated with host species in *Pinus* subsection *Strobus*.

Nevertheless, host association alone should not be regarded as a diagnostic character, as apparent host specificity may partly reflect sampling bias. Instead, host information is best combined with morphological and molecular evidence. Additional collections from a broader range of *Pinus* species are required to test whether the observed host specificity represents true biological specialization or a currently incomplete sampling of host range.

### Key to species of *Lophodermium* on pinus worldwide

**Table:** 

1	Ascomata subhypodermal	**2**
–	Ascomata otherwise embedded	**9**
2	Ascomata completely subhypodermal	**3**
–	Ascomata partly subhypodermal	**4**
3	Large ascomata (1.2–2.5 × 0.7–1.0 mm), basal stroma present, paraphyses strongly swollen at tips	***L. maximum***
–	Small ascomata (1–1.2 × 0.4–0.5 mm), basal stroma absent, paraphyses slightly swollen at tips	***L. dilutum***
4	Ascomata subhypodermal centrally, subepidermal at edge	***L. durilabrum***
–	Ascomata otherwise embedded	**5**
5	Lips absent	**6**
–	Lips present	**7**
6	Perimeter line present, black, basal stroma poorly developed	***L. ellipticum***
–	Perimeter line absent, basal stroma absent	***L. griseum***
7	Paraphyses strongly swollen at tips, zone line absent	***L. yanglingense***
–	Paraphyses slightly swollen at tips, black zone lines present	**8**
8	When dry, black in center for half of the total ascomata surface, shorter ascospores (40–80 µm)	***L. guangxiense***
–	When dry, black in center for a quarter of the total ascomata surface, longer ascospores (70–100 µm)	***L. indianum***
9	Completely subepidermal	**10**
–	Partly subepidermal or subcuticular	**24**
10	Basal stroma absent	**11**
–	Basal stroma present	**13**
11	Lips present, hyaline	***L. canberrianum***
–	Lips absent	**12**
12	When wet, ascomata dark brown, when dry pale brown, ascospores wider (2–4 µm)	***L. sichuanense***
–	When wet, ascomata black and shiny, when dry gray for a small region around the split, ascospores narrower (2 µm)	***L. orientale***
13	Basal stroma well developed	**14**
–	Basal stroma poorly developed	**16**
14	Paraphyses spiral at tips	***L. plumbeum***
–	Paraphyses not spiral at tips	**15**
15	Lips absent	***L. baculiferum***
–	Lips present	***L. pini-excelsae***
16	Lips yellow to orange	**17**
–	Lips other colors, or absent	**18**
17	Paraphyses branched at tips	***L. flavilabioides***
–	Paraphyses not branched at tips	***L. flavilabium***
18	Ascospores 1 septate	***L. yuexiense***
–	Ascospores aseptate	**19**
19	On/mostly on the abaxial side of needles	**20**
–	On both side of needles	**23**
20	Covering stroma extending to basal stroma	***L. resinosum***
–	Covering stroma not extending to basal stroma	**21**
21	Ascomata more or less orbicular, strongly raising above the surface of the substrate, perimeter line conspicuous	***L. puerense***
–	Ascomata elliptical, slightly or not raising above the surface of the substrate, perimeter line inconspicuous	**22**
22	Lips absent, with smaller asci (65–105 × 7–11 µm) and shorter (85–90 µm) ascospores	***L. pini-pumilae***
–	Lips present, with larger asci (140–170 × 11–14 µm) and longer (90–120 µm) ascospores	***L. seditiosum***
23	Longer ascomata (750–1100 µm), covering stroma extending to basal stroma, larger ascospores (70–100 × 2 µm)	***L. ravenelii***
–	Shorter ascomata (420–750 µm), covering stroma not extending to basal stroma, smaller ascospores (65–90 × 1–1.5 µm)	***L. haploxyli***
24	Ascomata partly subepidermal	**25**
–	Ascomata subcuticular	**41**
25	When dry, ascomata appearing as a thin black line between two rows of stomata, sometimes with a faint gray surround	***L. australe***
–	When dry, ascomata elliptical	**26**
26	Perimeter line absent	**27**
–	Perimeter line present	**30**
27	Basal stroma well developed	**28**
–	Basal stroma poorly developed	**29**
28	Ascomata narrower (200–250 µm), ends usually acute, lips present	***L. kumaunicum***
–	Ascomata wider (400–500 µm), ends rounded, lips absent	***L. pini-hwangshanensis***
29	Lips absent, paraphyses swollen at tips	***L. piceum***
–	Lips present, paraphyses not swollen at tips	***L. laojunshanense***
30	Basal stroma well developed	**31**
–	Basal stroma poorly developed	**34**
31	Paraphyses circinate at tips, intertwining with each other	***L. pini-taiwanensis***
–	Paraphyses not circinate at tips	**32**
32	Lip cells conspicuous, up to 22 µm long, paraphyses swollen up to 6 µm at tips	***L. corconticum***
–	Lip cells less than 22 µm, paraphyses slightly swollen at tips	**33**
33	Broader asci (14–20 µm) and ascospores (4 µm)	***L. iwatense***
–	Narrower asci (7–11 µm) and ascospores (2 µm)	***L. mirabile***
34	When dry, black in the center for less than a quarter of the total ascomata surface	***L. conigenum***
–	When dry, black in the center for more than a quarter of the total ascomata surface	**35**
35	Lips conspicuous	**36**
–	Lips inconspicuous	**37**
36	Paraphyses with a cylindrical swollen region at tips about 10 µm long	***L. himalayense***
–	Paraphyses not with a cylindrical swollen region at tips	***L. parasiticum***
37	Zone line rare or absent	**38**
–	Zone line abundant	**40**
38	Conidiomata absent	***L. macci***
–	Conidiomata present	**39**
39	Paraphyses swollen at tips, shorter conidia (4–6.5 µm)	***L. pini-bungeanae***
–	Paraphyses not swollen at tips, longer conidia (8–12 µm)	***L. staleyi***
40	Longer asci (110–155 µm), larger ascospores (70–110 × 2 µm)	***L. pinastri***
–	Shorter asci (65–80 µm), smaller ascospores (55–70 × 1 µm)	***L. huangshanense***
41	Ascomata elliptical or oblong-elliptical and arranged in a row along the median line of the needle surface, often several confluent to form linear structures	***L. confluens***
–	Ascomata not confluent	**42**
42	Covering stroma folded inwards	**43**
–	Covering stroma not folded inwards	**44**
43	Paraphyses swollen at tips, longer asci (85–100 µm) and ascospores (140–160 µm)	***L. fissuratum***
–	Paraphyses not swollen at tips, shorter asci (45–90 µm) and ascospores (40–80 µm)	***L. calceolatum***
44	Basal stroma well developed	**45**
–	Basal stroma poorly developed or absent	**46**
45	Lip cells well developed	***L. molitoris***
–	Lip cells poorly developed	***L. nitens***
46	Lip cells red brown	***L. anhuiense***
–	Lip cells hyaline or absent	**47**
47	Lip cells branched and septate, embedded in a gelatinous matrix	***L. pini-mugonis***
–	Lip cells not branched or absent	**48**
48	Distinct hyaline zone near the opening of covering stroma, zone line not seen	***L. jingpohuense***
–	No such structure, zone line abundant	***L. pini-sibiricae***

## Supplementary Material

XML Treatment for Lophodermium calceolatum

XML Treatment for Lophodermium flavilabium

XML Treatment for Lophodermium flavilabioides

XML Treatment for Lophodermium haploxyli

XML Treatment for Lophodermium huangshanense

XML Treatment for Lophodermium jingpohuense

XML Treatment for Lophodermium laojunshanense

XML Treatment for Lophodermium piceum

XML Treatment for Lophodermium pini-hwangshanensis

XML Treatment for Lophodermium plumbeum

## Additional information

### Use of AI

No use of AI was reported.

### Adherence to national and international regulations

All the fungal strains used in this study have been legally obtained, respecting the Convention on Biological Diversity (Rio Convention).

### Author ORCIDs

Shi-Juan Wang https://orcid.org/0009-0004-1711-4394

Lan Zhuo https://orcid.org/0000-0002-8129-2812

Xin-Yi Xu https://orcid.org/0009-0007-5689-2828

Xiao-Nan Sui https://orcid.org/0009-0005-3840-3969

Xiao-Ye Shen https://orcid.org/0000-0003-0789-6850

Yuan-Yuan Li https://orcid.org/0000-0002-6888-6800

Cheng-Lin Hou https://orcid.org/0000-0001-8162-5560

### Data availability

All of the data that support the findings of this study are available in the main text or Supplementary Information.

## Supplementary materials

Supplementary material 1 Phylogenetic tree generated by ML analysis based on sequences of the ITS https://doi.org/10.3897/imafungus.17.175730.suppl1 Data type jpg Explanation note *Cudoniella clavus* (AFTOL-ID 166) and Pezicula carpinea (KUS-F51029) were selected as outgroups. Maximum likelihood bootstrap values (MLB ≥ 75%) were shown at the nodes. https://binary.pensoft.net/file/1516001This dataset is made available under the Open Database License (http://opendatacommons.org/licenses/odbl/1.0/). The Open Database License (ODbL) is a license agreement intended to allow users to freely share, modify, and use this Dataset while maintaining this same freedom for others, provided that the original source and author(s) are credited. Shi-Juan Wang, Lan Zhuo, Xin-Yi Xu, Xiao-Nan Sui, Xiao-Ye Shen, Yuan-Yuan Li, Cheng-Lin Hou

Supplementary material 2 Phylogenetic tree generated by ML analysis based on sequences of the LSU and mtSSU https://doi.org/10.3897/imafungus.17.175730.suppl2 Data type jpg Explanation note *Cudoniella clavus* (AFTOL-ID 166) and Pezicula carpinea (KUS-F51029) were selected as outgroups. Maximum likelihood bootstrap values (MLB ≥ 75%) were shown at the nodes. https://binary.pensoft.net/file/1516002This dataset is made available under the Open Database License (http://opendatacommons.org/licenses/odbl/1.0/). The Open Database License (ODbL) is a license agreement intended to allow users to freely share, modify, and use this Dataset while maintaining this same freedom for others, provided that the original source and author(s) are credited. Shi-Juan Wang, Lan Zhuo, Xin-Yi Xu, Xiao-Nan Sui, Xiao-Ye Shen, Yuan-Yuan Li, Cheng-Lin Hou

Supplementary material 3 Taxa of *Rhytismatales* used for the phylogenetic analysis https://doi.org/10.3897/imafungus.17.175730.suppl3 Data type docx Explanation note Taxa of *Rhytismatales* used for the phylogenetic analysis, GenBank accession numbers, and voucher information. The newly generated sequences in the context of the present study are indicated in bold. https://binary.pensoft.net/file/1516003This dataset is made available under the Open Database License (http://opendatacommons.org/licenses/odbl/1.0/). The Open Database License (ODbL) is a license agreement intended to allow users to freely share, modify, and use this Dataset while maintaining this same freedom for others, provided that the original source and author(s) are credited. Shi-Juan Wang, Lan Zhuo, Xin-Yi Xu, Xiao-Nan Sui, Xiao-Ye Shen, Yuan-Yuan Li, Cheng-Lin Hou

Supplementary material 4 Alignment file https://doi.org/10.3897/imafungus.17.175730.suppl4 Data type fasta https://binary.pensoft.net/file/1516004This dataset is made available under the Open Database License (http://opendatacommons.org/licenses/odbl/1.0/). The Open Database License (ODbL) is a license agreement intended to allow users to freely share, modify, and use this Dataset while maintaining this same freedom for others, provided that the original source and author(s) are credited. Shi-Juan Wang, Lan Zhuo, Xin-Yi Xu, Xiao-Nan Sui, Xiao-Ye Shen, Yuan-Yuan Li, Cheng-Lin Hou
