# Supplementary figures and images for: Ten new species of Lophodermium (Rhytismatales, Rhytismataceae) on pine needles in China
Source: IMA Fungus. 2026 Jan 20;17:e175730. doi: 10.3897/imafungus.17.175730 (PMC12848512; doi:10.3897/imafungus.17.175730)

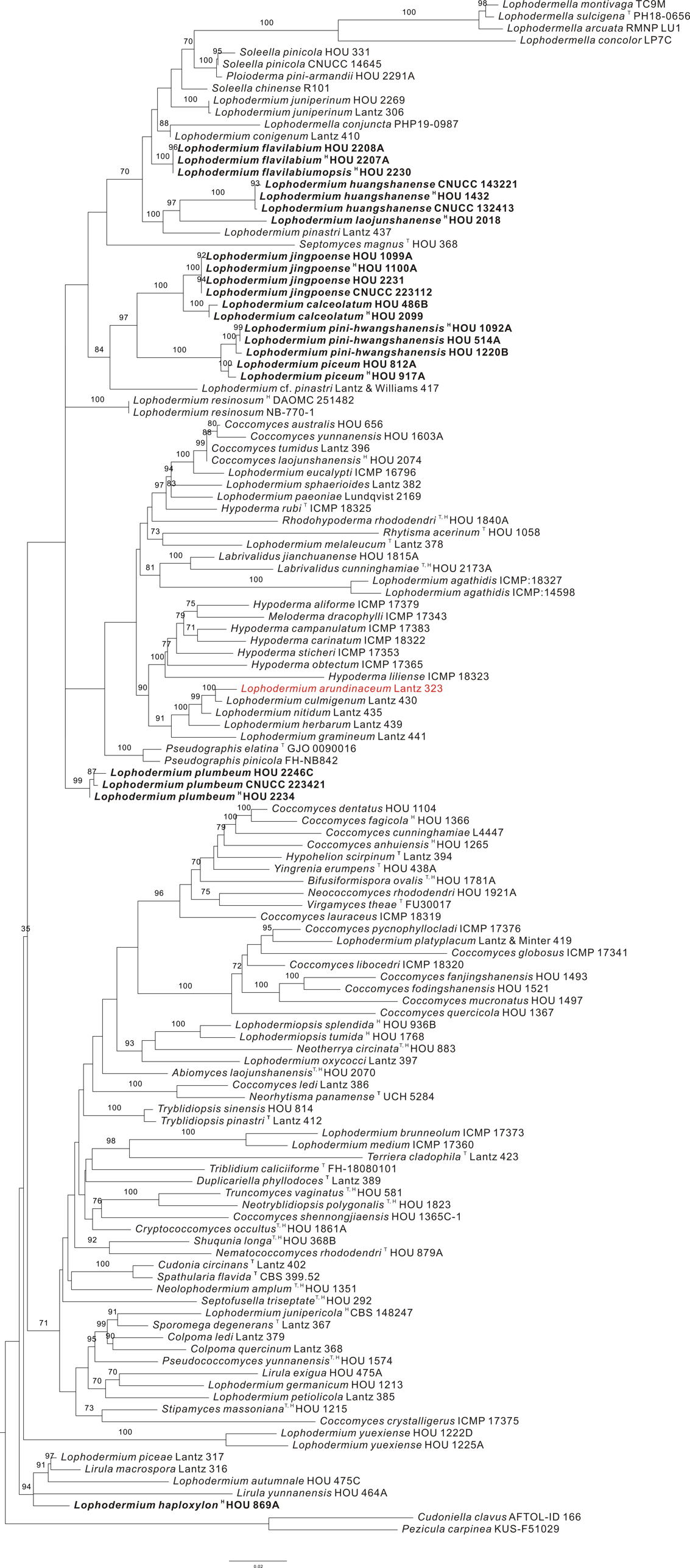

Supplement: Supplementary material 2 — Phylogenetic tree generated by ML analysis based on sequences of the LSU and mtSSU [file imafungus-17-e175730-s002.jpg]
