# Supplementary material for: Ten new species of Lophodermium (Rhytismatales, Rhytismataceae) on pine needles in China
Source: IMA Fungus. 2026 Jan 20;17:e175730. doi: 10.3897/imafungus.17.175730 (PMC12848512; doi:10.3897/imafungus.17.175730)
Supplement: Supplementary material 3 — Taxa of Rhytismatales used for the phylogenetic analysis [file imafungus-17-e175730-s003.docx]

Supplementary Table 1. Taxa of *Rhytismatales* used for the phylogenetic analysis, GenBank accession numbers, and voucher information. The newly generated sequences in the context of the present study are indicated in **bold**.

| **Species** | **Voucher** | **ITS** | **nrLSU** | **mtSSU** | **References** |
| --- | --- | --- | --- | --- | --- |
| *Abiomyces laojunshanensis* | HOU 2070 | PQ618625 | PQ618716 | PQ618762 | Zhuo et al. (2025a) |
| *Bifusella linearis* ^T^ | EBJul30-5 | KT000193 | — | — | Kirk et al. (2015) |
| *Bifusiformispora ovalis* ^T, H^ | HOU 1781A | PV598081 | PV598105 | PV598130 | Zhuo et al. (2025b) |
| *Bivallum zelandicum* ^T^ | PDD 112248 | MH921846 | — | — | Johnston et al. (2019) |
| *Coccomyces anhuiensis* ^H^ | HOU 1265 | MK371313 | MK371314 | MK371315 | Lv and Hou (2019) |
| *Coccomyces australis* | HOU 656 | KF797434 | KF797446 | KF797457 | Wang et al. (2014b) |
| *Coccomyces circinatus* | T 72 | GU138722 | — | — | GenBank (2025) |
| *Coccomyces clavatus* | PDD 112233 | MH578536 | — | — | Lv et al. (2019) |
| *Coccomyces crystalligerus* | ICMP 17375 | — | HM140501 | HM143778 | Lantz et al. (2011) |
| *Coccomyces cunninghamiae* | L4447 | OR669146 | OR669148 | OR669150 | Zheng et al. (2024) |
| *Coccomyces delta* | R 68 | GU138731 | — | — | Lv et al. (2019) |
| *Coccomyces dentatus* | HOU 1104 | KF797433 | KF797445 | KF797456 | Wang et al. (2014b) |
| *Coccomyces fagicola* ^H^ | HOU 1366 | MN385774 | MN372085 | MN385767 | Lv et al. (2019) |
| *Coccomyces fanjingshanensis* | HOU 1493 | MT123016 | MT122763 | MT123012 | Zang et al. (2020) |
| *Coccomyces globosus* | ICMP 17341 | — | HM140503 | HM143780 | Lantz et al. (2011) |
| *Coccomyces huangshanensis* | R86c | GU138733 | — | — | Lv et al. (2019) |
| *Coccomyces lauraceus* | ICMP 18319 | — | HM140504 | HM143781 | Lantz et al. (2011) |
| *Coccomyces ledi* | Lantz 386 (UPS) | — | HM140505 | HM143782 | Lantz et al. (2011) |
| *Coccomyces libocedri* | ICMP 18320 | — | HM140507 | HM143784 | Lantz et al. (2011) |
| *Coccomyces mucronatus* | HOU 1497 | PP488559 | PP488671 | PP488766 | Guo et al. (2024) |
| *Coccomyces pycnophyllocladi* | ICMP 17376 | NR_175748 | NG_081313 | — | Crous et al. (2021) |
| *Coccomyces quercicola* | HOU 1367 | MN385775 | MN372090 | MN385765 | Lv et al. (2019) |
| *Coccomyces shennongjiaensis* | HOU 1365C.1 | MN385776 | MN385769 | MN385768 | Lv et al. (2019) |
| *Coccomyces sinensis* | Cj01 | AB787513 | — | — | Matsukura et al. (2017) |
| *Coccomyces triangularis* | MUOB 366039 | OK376742 | — | — | GenBank (2025) |
| *Coccomyces tumidus* ^T^ | Lantz 396 | — | HM140510 | HM143787 | Lantz et al. (2011) |
| *Coccomyces yunnanensis* | HOU 1603A | PP488560 | — | PP488767 | Guo et al. (2024) |
| *Colpoma ledi* | Lantz 379 | — | HM140512 | HM143788 | Lantz et al. (2011) |
| *Colpoma quercinum* ^T^ | Lantz 368 | — | HM140513 | HM143789 | Lantz et al. (2011) |
| *Cryptococcomyces occultus* ^T, H^ | HOU 1861A | PQ618635 | PQ618726 | PQ618772 | Zhuo et al. (2025a) |
| *Cudonia circinans* ^T^ | Lantz 402 | — | HM140515 | HM143791 | Lantz et al. (2011) |
| *Cudoniella clavus* | AFTOL-ID 166 | DQ491502 | DQ470944 | FJ713604 | Spatafora et al. (2006) |
| *Davisomycella medusa* | BPI842078 | AY465525 | — | — | Ganley et al. (2004) |
| *Elytroderma deformans* ^T^ | CBS 181.68 | AF203469 | — | — | Ortiz-García et al. (2003) |
| *Hypoderma aliforme* ^H^ | ICMP 17379 | NR_175749 | NG_081314 | — | Crous et al. (2021) |
| *Hypoderma campanulatum* | ICMP 17383 | — | HM140517 | HM143792 | Lantz et al. (2011) |
| *Hypoderma caricis* | R 17 | GU138752 | — | — | Wang et al. (2013) |
| *Hypoderma carinatum* | ICMP 18322 | — | HM140518 | HM143793 | Lantz et al. (2011) |
| *Hypoderma liliense* | ICMP 18323 | — | HM140523 | HM143798 | Lantz et al. (2011) |
| *Hypoderma obtectum* | ICMP 17365 | — | HM140525 | HM143800 | Lantz et al. (2011) |
| *Hypoderma rubi* ^T^ | ICMP 18325 | — | HM140527 | HM143802 | Lantz et al. (2011) |
| *Hypoderma siculum* | PDD 99894 | JF683424 | — | — | Lantieri et al. (2012) |
| *Hypohelion scirpinum* ^T^ | Lantz 394 | — | HM140531 | HM143806 | Lantz et al. (2011) |
| *Labivalidus cunninghamiae* ^H^ | HOU 2173A | PQ618642 | PQ618732 | PQ618778 | Zhuo et al. (2025a) |
| *Labivalidus jianchuanensis* | HOU 1815A | PQ618646 | PQ618734 | — | Zhuo et al. (2025a) |
| *Lirula exigua* | HOU 475A | HQ902157 | HQ902150 | — | Fan et al. (2012) |
| *Lirula macrospora* | Lantz 316 | — | — | HM143807 | Lantz et al. (2011) |
| *Lirula yunnanensis* | HOU 464A | HQ902156 | HQ902149 | — | Fan et al. (2012) |
| *Lophodermella arcuata* | RMNP LU1 | MN937644 | MN937585 | — | Ata et al. (2021) |
| *Lophodermella concolor* | LP7C | MN937621 | MN937588 | — | Ata et al. (2021) |
| *Lophodermella conjuncta* | PHP19-0987 | MN937608 | MN937603 | — | Ata et al. (2021) |
| *Lophodermella montivaga* | TC9M | MN937629 | MN937599 | — | Ata et al. (2021) |
| *Lophodermella sulcigena* ^T^ | PH18-0656 | MN937624 | MN937604 | — | Ata et al. (2021) |
| *Lophodermiopsis splendida* ^H^ | HOU 936B | PP488570 | PP488680 | PP488773 | Guo et al. (2024) |
| *Lophodermiopsis tumida* ^H^ | HOU 1768 | PP488572 | PP488682 | PP488775 | Guo et al. (2024) |
| *Lophodermium actinothyrium* | losa139 | AY100663 | — | — | Ortiz-García et al. (2003) |
| *Lophodermium agathidis* | ICMP:18327 | — | HM140534 | HM143810 | Lantz et al. (2011) |
| *Lophodermium agathidis* | ICMP:14598 | — | HM140532 | HM143808 | Lantz et al. (2011) |
| *Lophodermium arundinaceum* ^T^ | Lantz 323 | — | HM140535 | HM143811 | Lantz et al. (2011) |
| *Lophodermium australe* | isolate 24 | EU696778 | — | — | Toju et al. (2013) |
| *Lophodermium autumnale* | HOU 475C | HQ902158 | HQ902151 | — | Fan et al. (2012) |
| *Lophodermium baculiferum* | mon2zem Nuevo Leon | AY100656 | — | — | Ortiz-García et al. (2003) |
| *Lophodermium baculiferum* | loba1 Oregon | AY100655 | — | — | Ortiz-García et al. (2003) |
| *Lophodermium brunneolum* | ICMP 17373 | — | HM140536 | HM143812 | Lantz et al. (2011) |
| ***Lophodermium calceolatum*** | **CNUCC 209911** | **PX696040** | **—** | **—** | **This study** |
| ***Lophodermium calceolatum*** | **HOU 486B** | **PX731546** | **PX732845** | **PX613967** | **This study** |
| ***Lophodermium calceolatum* ^H^** | **HOU 2099** | **PX731547** | **PX732846** | **—** | **This study** |
| *Lophodermium cathayae* | Hou 831M | HQ992812 | — | — | Gao et al. (2013) |
| *Lophodermium cedrinum* | BCAB212 | MG779472 | — | — | GenBank (2025) |
| *Lophodermium cephalotaxi* | T15 | GU138701 | — | — | GenBank (2025) |
| *Lophodermium conigenum* | Lantz 410 (UPS) | — | HM140537 | HM143813 | Lantz et al. (2011) |
| *Lophodermium corconticum* | SY370 | OK493168 | — | — | Lantz et al. (2011) |
| *Lophodermium culmigenum* | Lantz 430 (UPS) | — | HM140539 | HM143815 | Lantz et al. (2011) |
| *Lophodermium eucalypti* | ICMP 16796 | — | HM140541 | HM143817 | Lantz et al. (2011) |
| *Lophodermium fissuratum* | CA2PM1-19-1 | KY576859 | — | — | Salas‐Lizana and Oono (2018) |
| *Lophodermium fissuratum* | CA2PM1-14-1 | KY576858 | — | — | Salas‐Lizana & Oono (2018) |
| ***Lophodermium flavilabium*** | **HOU 2208A** | **PX731548** | **PX732847** | **PX613968** | **This study** |
| ***Lophodermium flavilabium*** | **CNUCC 2207A11** | **PX696041** | **—** | **—** | **This study** |
| ***Lophodermium flavilabium*** | **CNUCC 2208A11** | **PX696042** | **—** | **—** | **This study** |
| ***Lophodermium flavilabium* ^H^** | **HOU 2207A** | **PX731549** | **PX732848** | **PX613969** | **This study** |
| ***Lophodermium flavilabiumopsis*** | **CNUCC 223011** | **PX696043** | **—** | **—** | **This study** |
| ***Lophodermium flavilabiumopsis*** | **CNUCC 2249B21** | **PX696044** | **—** | **—** | **This study** |
| ***Lophodermium flavilabiumopsis* ^H^** | **HOU 2230** | **PX731550** | **—** | **PX613970** | **This study** |
| *Lophodermium gamundiae* ^H^ | ICMP 16802 | NR_119628 | — | — | GenBank (2025) |
| *Lophodermium germanicum* | HOU 1213 | — | PV598108 | PV598133 | Zhuo et al. (2025b) |
| *Lophodermium gramineum* | Lantz 441 (UPS) | — | HM140542 | — | Lantz et al. (2011) |
| ***Lophodermium haploxylon*** | **HOU 2249A** | **PX731551** | **—** | **—** | **This study** |
| ***Lophodermium haploxylon*** | **CNUCC 2249A11** | **PX696045** | **—** | **—** | **This study** |
| ***Lophodermium haploxylon*** | **HOU 2257B** | **PX731552** | **—** | **—** | **This study** |
| ***Lophodermium haploxylon* ^H^** | **HOU 869A** | **PX731553** | **PX732849** | **PX613971** | **This study** |
| *Lophodermium harbinense* | HOU 2264 | PV598085 | — | — | Zhuo et al. (2025b) |
| *Lophodermium hauturuanum* | PDD 112227 | MH578524 | — | — | Johnston et al. (2019) |
| *Lophodermium herbarum* | Lantz 439 (UPS) | — | HM140543 | HM143818 | Lantz et al. (2011) |
| ***Lophodermium huangshanense*** | **CNUCC 143221** | **PX696046** | **PX732863** | **PX613965** | **This study** |
| ***Lophodermium huangshanense*** | **CNUCC 132413** | **PX696047** | **PX732864** | **PX613966** | **This study** |
| ***Lophodermium huangshanense*** | **HOU 1221** | **PX731554** | **—** | **—** | **This study** |
| ***Lophodermium huangshanense* ^H^** | **HOU 1432** | **PX731555** | **PX732850** | **PX613972** | **This study** |
| *Lophodermium implicatum* | T9 | GU138700 | — | — | GenBank (2025) |
| *Lophodermium indianum* | CMW 39125 | KF636510 | — | — | Tanney and Seifert (2017) |
| *Lophodermium jiangnanense* | Cj12 | AB787524 | — | — | Matsukura et al. (2017) |
| ***Lophodermium jingpoense*** | **HOU 1099A** | **PX731556** | **PX732851** | **PX613973** | **This study** |
| ***Lophodermium jingpoense*** | **HOU 2231** | **PX731557** | **PX732852** | **—** | **This study** |
| ***Lophodermium jingpoense*** | **CNUCC 223112** | **PX696048** | **PX732865** | **—** | **This study** |
| ***Lophodermium jingpoense*** | **HOU 2209** | **PX731558** | **—** | **—** | **This study** |
| ***Lophodermium jingpoense* ^H^** | **HOU 1100A** | **PX731559** | **PX732853** | **PX613974** | **This study** |
| *Lophodermium johnstonii* | isolate 310 | JX232417 | — | — | Wang et al. (2013) |
| *Lophodermium junipericola* | CBS 148247 | NR_175216 | NG_081325 | — | Crous et al. (2021) |
| *Lophodermium juniperinum* | Lantz 306 (UPS) | — | HM140544 | HM143819 | Lantz et al. (2011) |
| *Lophodermium juniperinum* | HOU 2269 | PV598087 | PV598110 | PV598135 | Zhuo et al. (2025b) |
| *Lophodermium kumaunicum* | 21 | EU696776 | — | — | GenBank (2025) |
| *Lophodermium kumaunicum* | 22 | EU696777 | — | — | GenBank (2025) |
| *Lophodermium macci* | wxm130 | HM037981 | — | — | GenBank (2025) |
| *Lophodermium medium* | ICMP 17360 | — | HM140545 | HM143820 | Lantz et al. (2011) |
| *Lophodermium melaleucum* | Lantz 378 (UPS) | — | HM140546 | HM143821 | Lantz et al. (2011) |
| *Lophodermium molitoris* | CBS 597.84 | AY100659 | — | — | Ortiz-García et al. (2003) |
| *Lophodermium molitoris* | SJV2_10a | KM106818 | — | — | Oono et al. (2014) |
| *Lophodermium nitens* | Sie1PL2-14-1 | MG877536 | — | — | Salas‐Lizana and Oono (2018) |
| *Lophodermium nitens* | SA23221 | MG877437 | — | — | Salas‐Lizana & Oono (2018) |
| *Lophodermium nitidum* | Lantz 435 (UPS) | — | HM140547 | HM143822 | Lantz et al. (2011) |
| *Lophodermium oxycocci* | Lantz 397 (UPS) | — | — | HM143823 | Lantz et al. (2011) |
| *Lophodermium paeoniae* | Lundqvist 21693 | — | HM140549 | — | Lantz et al. (2011) |
| *Lophodermium petiolicola* | Lantz 385 (UPS) | — | HM140550 | HM143824 | Lantz et al. (2011) |
| *Lophodermium petrakii* | R60 | FJ861984 | — | — | Tanney and Seifert (2017) |
| *Lophodermium piceae* | Lantz 317 (UPS) | — | HM140551 | HM143825 | Lantz et al. (2011) |
| ***Lophodermium piceum*** | **HOU 1948** | **PX731560** | **—** | **—** | **This study** |
| ***Lophodermium piceum*** | **CNUCC 2299B11** | **PX696049** | **—** | **—** | **This study** |
| ***Lophodermium piceum*** | **HOU 812A** | **PX731561** | **PX732854** | **PX613975** | **This study** |
| ***Lophodermium piceum* ^H^** | **HOU 917A** | **PX731562** | **PX732855** | **PX613976** | **This study** |
| *Lophodermium pinastri* | Lantz 437 | — | HM140552 | HM143826 | Lantz et al. (2011) |
| *Lophodermium* cf. *pinastri* | Lantz & Williams 417 (UPS) | — | HM140553 | — | Lantz et al. (2011) |
| *Lophodermium pini-bungeanae* | R112 | FJ861989 | — | — | Tanney and Seifert (2017) |
| *Lophodermium pini-excelsae* | R85 | FJ861987 | — | — | Tanney and Seifert (2017) |
| ***Lophodermium pini-hwangshanensis*** | **HOU 1919** | **PX731563** | **—** | **—** | **This study** |
| ***Lophodermium pini-hwangshanensis*** | **HOU 514A** | **PX731564** | **—** | **PX613977** | **This study** |
| ***Lophodermium pini-hwangshanensis*** | **HOU 1220B** | **PX731565** | **PX732856** | **PX613978** | **This study** |
| ***Lophodermium pini-hwangshanensis* ^H^** | **HOU 1092A** | **PX731566** | **PX732857** | **PX613979** | **This study** |
| *Lophodermium pini-mugonis* | 568M | JF332165 | — | — | Hou et al. (2009) |
| *Lophodermium pini-taiwanensis* | HOU 1222B | KX443656 | — | — | Li et al. (2016) |
| *Lophodermium platyplacum* | Lantz & Minter 419 (UPS) | — | HM140554 | HM143827 | Lantz et al. (2011) |
| ***Lophodermium plumbeum*** | **HOU 2246C** | **PX731567** | **PX732858** | **—** | **This study** |
| ***Lophodermium plumbeum*** | **CNUCC 223421** | **PX696050** | **PX732866** | **—** | **This study** |
| ***Lophodermium plumbeum*** | **CNUCC 2246C21** | **PX696051** | **—** | **—** | **This study** |
| ***Lophodermium plumbeum*** | **HOU 2234** | **PX731568** | **PX732859** | **—** | **This study** |
| *Lophodermium rectangulare* | PDD 112235 | MH578540 | — | — | Johnston et al. (2019) |
| *Lophodermium resinosum* | NB-770-1 | KY485129 | KY485135 | — | Tanney & Seifert (2017) |
| *Lophodermium resinosum* ^H^ | DAOMC 251482 | NR_172257 | NG_060349 | — | Tanney and Seifert (2017) |
| *Lophodermium seditiosum* | SY366 | OK493165 | — | — | GenBank (2025) |
| *Lophodermium sphaerioides* | Lantz 382 (UPS) | — | HM140556 | HM143829 | Lantz et al. (2011) |
| *Lophodermium thailandicum* | MFLU 17-0673 | MG821634 | — | — | Hyde et al. (2018) |
| *Lophodermium tindalii* | PDD 92044 | MH921867 | — | — | Johnston et al. (2019) |
| ***Lophodermium yuexiense*** | **HOU 1222D** | **PX731569** | **PX732860** | **PX613980** | **This study** |
| ***Lophodermium yuexiense*** | **HOU 1225A** | **PX731570** | **PX732861** | **PX613981** | **This study** |
| *Meloderma desmazieri* ^T^ | MD3 (ATCCc) | AF426056 | — | — | Deckert et al. (2002) |
| *Meloderma dracophylli* | ICMP 17343 | — | HM140561 | HM143833 | Lantz et al. (2011) |
| *Nematococcomyces rhododendri* ^T^ | HOU 879A | — | KC312685 | KC312690 | Tian et al. (2013) |
| *Neococcomyces rhododendri* | HOU 1921A | PP488592 | PP488699 | PP488791 | Guo et al. (2024) |
| *Neorhytisma panamense* ^T^ | UCH 5284 | OQ944277 | — | OQ944356 | Wang et al. (2023) |
| *Neotherrya circinata* ^T, H^ | HOU 883 | PQ618651 | PQ618739 | PQ618783 | Zhuo et al. (2025a) |
| *Neotryblidiopsis polygonalis* ^T, H^ | HOU 1823 | PP488594 | PP488701 | PP488793 | Guo et al. (2024) |
| *Occultimyces fusiformis* ^T, H^ | HOU 458A | PP488603 | — | — | Guo et al. (2024) |
| *Pezicula carpinea* | KUS-F51029 | JN033388 | JN086691 | JN086765 | Han et al. (2014) |
| *Ploioderma destruens* | T33 | GU138756 | — | — | GenBank (2025) |
| ***Ploioderma pini-armandii*** | **HOU 2292C** | **PX731571** | **—** | **—** | **This study** |
| ***Ploioderma pini-armandii*** | **HOU 2291A** | **PX731572** | **PX732862** | **—** | **This study** |
| *Pseudococcomyces yunnanensis* ^T, H^ | HOU 1574 | PQ618656 | PQ618743 | PQ618788 | Zhuo et al. (2025a) |
| *Pseudographis elatina* ^T^ | GJO 0090016 | MK751794 | MK751803 | MK751717 | Karakehian et al. (2019) |
| *Pseudographis pinicola* | FH NB842 | MK751796 | MK751805 | MK751719 | Karakehian et al. (2019) |
| *Rhodohypoderma rhododendri* ^T, H^ | HOU 1840A | PP488608 | PP488712 | PP488806 | Guo et al. (2024) |
| *Rhytisma acerinum* ^T^ | HOU 1058 | OQ944148 | OQ944295 | OQ944332 | Guo et al. (2024) |
| *Septofusella triseptate* ^T, H^ | HOU 292 | PP488615 | PP488718 | PP488813 | Guo et al. (2024) |
| *Septomyces magnus* ^T^ | HOU 368 | PP488618 | PP488720 | — | Guo et al. (2024) |
| *Shuqunia longa* ^T, H^ | HOU 368B | PP488620 | PP488721 | PP488815 | Guo et al. (2024) |
| *Soleella chinense* | R101 | PV598088 | PV598111 | PV598136 | Zhuo et al. (2025b) |
| *Soleella pinicola* | HOU 331 | PV598089 | PV598112 | PV598137 | Zhuo et al. (2025b) |
| *Soleella pinicola* | CNUCC 14645 | PV598102 | PV598123 | PV598149 | Zhuo et al. (2025b) |
| *Spathularia flavida* ^T^ | CBS 399.52 | — | AY541496 | AY575101 | Lumbsch et al. (2005) |
| *Sporomega degenerans* ^T^ | Lantz 367 | — | HM140567 | HM143839 | Lantz et al. (2011) |
| *Stipamyces massonianae* ^T, H^ | HOU 1215 | PQ618657 | PQ618744 | PQ618789 | Zhuo et al. (2025a) |
| *Terriera cladophila* ^T^ | Lantz 423 | — | HM140568 | HM143840 | Lantz et al. (2011) |
| *Therrya eucalypti* | PRJ AU09-82 | KM880188 | — | — | GenBank (2025) |
| *Therrya pinicola* | TRY | MT707244 | — | — | Haelewaters et al. (2020) |
| *Therrya strobi* | NB-645C | MH457134 | — | — | McMullin et al. (2019) |
| *Triblidium caliciiforme* ^T^ | FH-18080101 | MK751798 | MK751807 | MK751721 | Karakehian et al. (2019) |
| *Tryblidiopsis pinastri* ^T^ | Lantz 412 | — | HM140573 | — | Lantz et al. (2011) |
| *Tryblidiopsis sinensis* ^H^ | HOU 814 | KC312674 | KC312681 | KC312694 | Wang et al. (2014a) |
| *Virgamyces theae* ^T^ | FU30017 | KF797432 | KF797444 | — | Wang et al. (2014b) |
| *Yingrenia erumpens* ^T^ | HOU 438A | PP488648 | PP488744 | PP488840 | Guo et al. (2024) |

Notes：HOU: Collecting number; CNUCC: Isolate number; “—” sequences are not available; Species name ^T^ – Type species; Species name ^H^ – Holotype
